# A Tumor-Microenvironment-Responsive Chemically Masked IL‑2 Prodrug Potentiates PD‑1 Checkpoint Blockade and Elicits Robust Antitumor Efficacy

**DOI:** 10.1021/acscentsci.6c00344

**Published:** 2026-08-05

**Authors:** Linzhi Tan, Yunwen Yang, Kerui Zhao, Jingjing Zhou, Xin Wang, Xingyu Zhao, Qiang Lu, Junyu Lin, Yijie Zhao, Yushun Tian, Yong Wang, Chao Zhong, Tao Liu

**Affiliations:** † State Key Laboratory of Natural and Biomimetic Drugs, Chemical Biology Center, Department of Molecular and Cellular Pharmacology, School of Pharmaceutical Sciences, Peking University Third Hospital, Institute of Advanced Clinical Medicine, Peking University, Beijing 100191, China; ‡ Department of Immunology, School of Basic Medical Sciences, NHC Key Laboratory of Medical Immunology, Medicine Innovation Center for Fundamental Researches on Major Immunology-Related Diseases, Peking University, Beijing 100191, China; § PKUHSC-Beijing Huida Cell Technology Co. Ltd. Joint Laboratory for Codon Innovation, Beijing 100191, China; ∥ Department of Laboratory Animal Science, Peking University Health Science Center, Beijing 100191, China; ⊥ Beijing Huida Cell Technology Co., Ltd., Beijing 100176, China

## Abstract

Interleukin-2 (IL-2) is a potent mediator of T-cell activation with significant potential for cancer immunotherapy, yet its clinical utility is severely constrained by its narrow therapeutic window. Here we report a chemically masked IL-2 prodrug (Cm-proIL2) that enables tumor-microenvironment-responsive cytokine activation through tumor-associated protease cleavage. Site-specific conjugation of a poly­(ethylene glycol) (PEG) moiety selectively masks peripheral receptor engagement, while PEG removal within the tumor microenvironment restores IL-2 receptor binding and reduces molecular size, thereby enhancing intratumoral lymphocyte penetration and effector T cell functionality. Building on this modular platform, we further engineered a PD-1-targeted nanobody fusion, PD1-Cm-proIL2, to enable the cis delivery of IL-2 activity to PD-1^+^ tumor-infiltrating T cells. PD1-Cm-proIL2 induces robust antitumor immunity, resulting in complete tumor regression and durable protection upon tumor rechallenge in a murine colorectal cancer model. Together, these findings demonstrate the feasibility of chemically masked, tumor-responsive cytokine activation as a strategy for improving the therapeutic index of IL-2-based immunotherapies.

## Graphical abstract

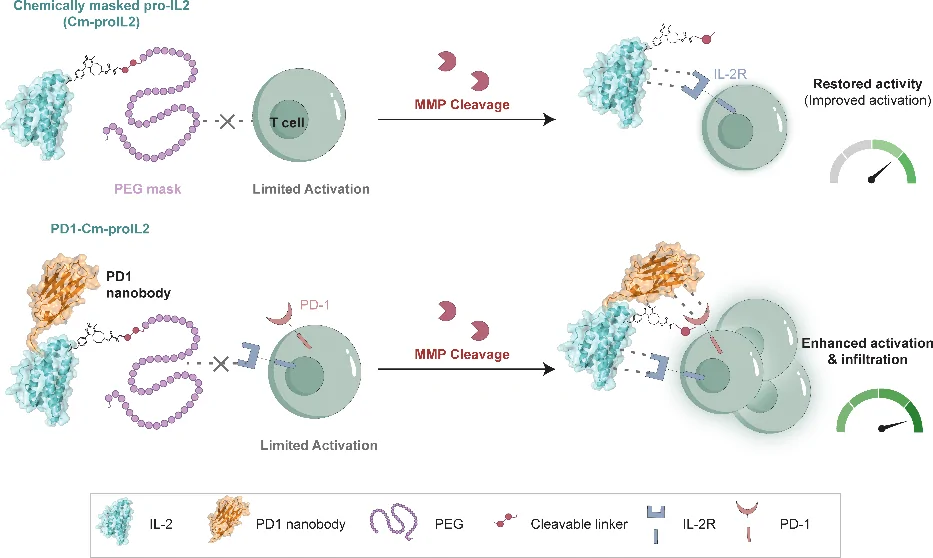

## Introduction

Cytokines hold substantial promise for cancer immunotherapy owing to their potent capacity to activate and expand immune effector cells;^1^ however, their clinical application has been broadly constrained by systemic toxicity and narrow therapeutic windows. Interleukin-2 (IL-2) is among the most extensively studied and clinically validated cytokines for cancer treatment,^2–5^ yet systemic administration triggers broad immune activation that restricts dose escalation and durable benefit. These challenges are rooted in the pleiotropic nature of IL-2 signaling, which is mediated by receptor complexes with varying affinities.^6,7^ While effector CD8^+^ T cells preferentially express the intermediate-affinity dimeric receptor (IL-2Rβγ), immunosuppressive regulatory T (Treg) cells constitutively express the high-affinity trimeric receptor (IL-2Rαβγ, including IL-2Rα/CD25). Consequently, systemic administration often leads to the preferential activation of Treg cells^8,9^ and vascular endothelial cells^10,11^ at low doses, whereas the higher concentrations required to stimulate antitumor effectors inevitably trigger systemic inflammatory syndromes and vascular leak.^12–14^ This receptor heterogeneity creates an inherent pharmacological trade-off, necessitating the development of engineering strategies capable of confining potent IL-2 activity to the tumor microenvironment (TME) while minimizing peripheral receptor engagement.

To improve the therapeutic index of IL-2, multiple engineering approaches have been explored, including receptor-biased variants,^15–22^ PEGylated IL-2 conjugates,^23–26^ and antibody-based masking.^27–29^ Although these designs have achieved improvements in safety or pharmacokinetics, many rely on permanent attenuation of cytokine activity or receptor engagement, thereby constraining maximal efficacy. To decouple systemic exposure from local immune activation without intrinsically weakening cytokine function, pro-cytokine strategies have been developed to enable conditional activation in defined biological contexts. Time-dependent chemical masking approaches, exemplified by bempegaldesleukin (NKTR-214),^24^ prolong circulation through hydrolytically labile PEGylation; however, activation is primarily dictated by systemic hydrolysis rather than tumor-derived cues, limiting tumor-selective activation of IL-2 signaling. In parallel, receptor-based pro-cytokine designs introduce tumor-conditional activation through engineered masking domains.^30–32^ However, incomplete unmasking after cleavage can leave residual attenuation of receptor engagement, and the mask geometry and linker design typically must be customized to each cytokine–receptor interface, limiting generalizability.

Here we report a chemically masked, tumor microenvironment-responsive IL-2 prodrug (Cm-proIL2) enabled by genetic code expansion (GCE)-mediated incorporation of a tetrazine-bearing noncanonical amino acid (ncAA) for inverse-electron-demand Diels–Alder (IEDDA) bioorthogonal conjugation to achieve site-specific PEG conjugation. In this design, PEG serves as a chemically orthogonal masking moiety that suppresses cytokine activity in peripheral tissues while allowing complete restoration upon proteolytic cleavage within the tumor microenvironment. Compared with an otherwise identical, noncleavable PEGylated IL-2, Cm-proIL2 elicited enhanced intratumoral lymphocyte infiltration and preserved effector T-cell functional states, resulting in superior antitumor efficacy. Building on recent advances in PD-1/IL-2 bispecifics,^21,33,34^ we further fused Cm-proIL2 to a PD-1-targeting nanobody to generate PD1-Cm-proIL2. This approach selectively redirects IL-2 activity toward PD-1^+^ T cells, promoting expansion of tumor-reactive CD8^+^ T cells while concurrently releasing the PD-1 checkpoint “brake”, thereby generating a potent synergy between checkpoint blockade and IL-2 stimulation. In the MC38 murine colorectal cancer model, PD1-Cm-proIL2 elicits robust antitumor activity, including complete tumor regressions and protection upon tumor rechallenge. Together, our findings provide proof of concept for combining tumor microenvironment-responsive cytokine activation with immune-cell targeting to enhance the antitumor activity of IL-2 while improving its therapeutic profile.

## Results

### Developing a TME-Responsive Chemically Masked IL-2 with Restored Bioactivity and Enhanced Tissue Penetrability

To develop Cm-proIL2 featuring site-specific PEG conjugation via GCE and enable complete activity recovery (Figure 1a), we first sought to identify a TME-responsive peptide linker. Because matrix metalloproteinases (MMPs) are abundantly expressed in the TME,^35–37^ we designed Cm-proIL2 with an MMP-responsive linker to enable selective restoration of IL-2Rα binding within tumors. To enable efficient PEG removal in the TME, we screened a panel of MMP-cleavable peptide sequences^30,32^ using MBP-peptide-sfGFP fusion proteins as a model system (Figure S1). Among the candidates, the MMP-2 substrate PLGLAG was selected owing to its synthetic accessibility and minimal residual sequence, leaving only three amino acids after cleavage,^38^ which facilitates near-complete recovery of receptor binding activity. Based on this design, a *trans*-cyclooctene (TCO)-modified, MMP-cleavable PEG reagent, TCO-GPLGLAGC-PEG20K, was synthesized for subsequent conjugation. In this architecture, the initial glycine and terminal cysteine residues were strategically incorporated to facilitate the attachment of the TCO handle and the PEG chain, respectively, while preserving the proteolytic sensitivity of the PLGLAG core (Figure S2a).

**Figure 1:**
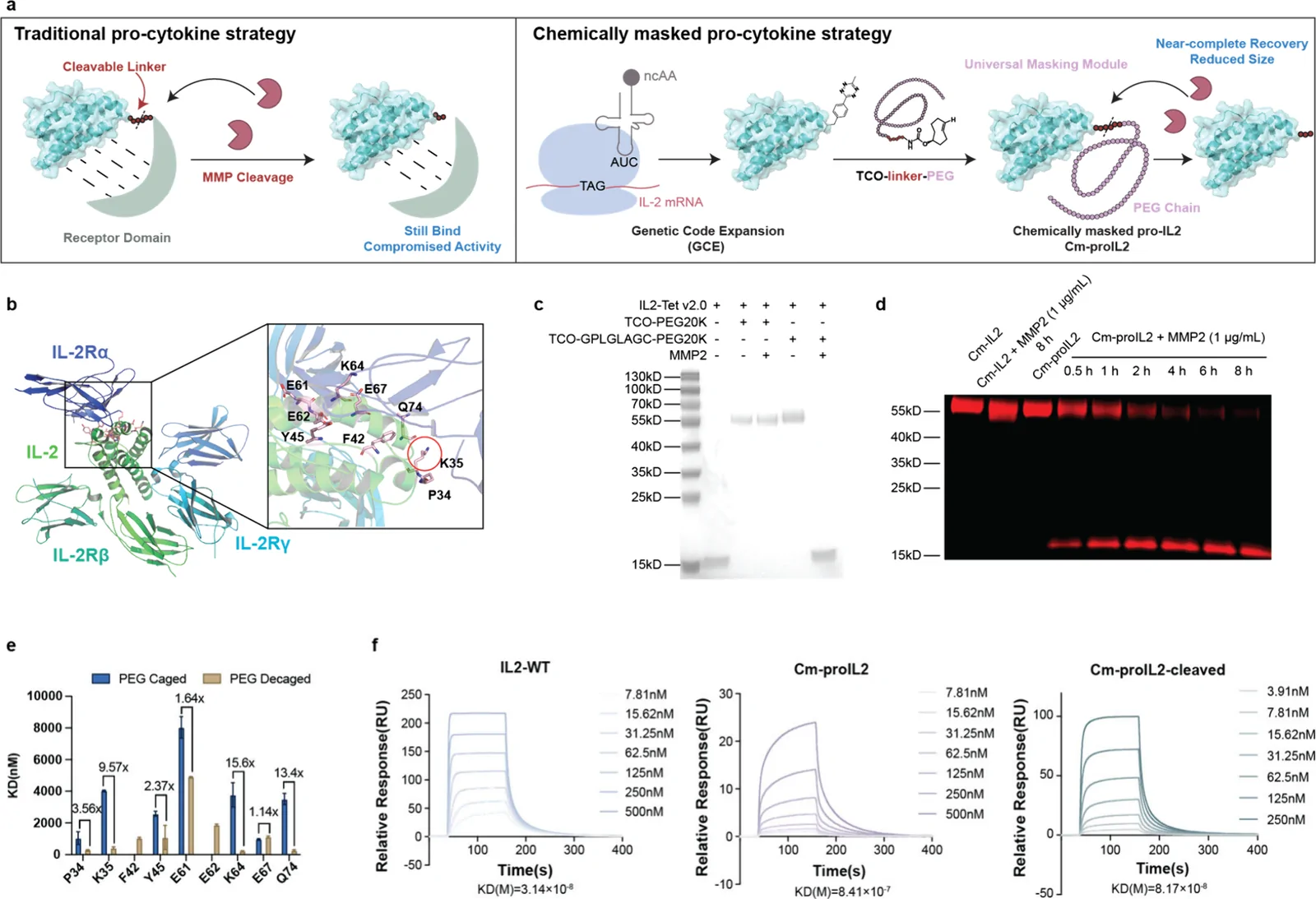
**Construction and characterization of Cm-proIL2**. (a) Conceptual framework for chemically masked pro-cytokine activation. (b) Candidate PEGylation sites mapped onto the crystal structure of the IL-2/IL-2R heterotrimeric complex (PDB entry 2ERJ). The K35 residue selected for proIL-2 engineering is indicated by a red circle. (c) SDS-PAGE analysis showing TCO-PEG20K or TCO-GPLGLAGC-PEG20K conjugated to Tet v2.0 incorporated IL-2 and the consequent MMP-2 (2 μg/mL) cleavage. (d) Time-dependent cleavage of Cm-proIL2 by MMP-2 (1 μg mL^–1^). (e) Comparison of murine IL-2Rα binding affinities for IL-2 variants PEGylated at different sites, measured in both the PEG-conjugated state and after MMP-mediated cleavage. F42- and E62-modified variants displayed minimal IL-2Rα binding. (f) Surface plasmon resonance analysis of wild-type IL-2 (IL2-WT), intact Cm-proIL2, and cleaved Cm-proIL2.

We next performed site screening to enable site-specific PEG conjugation. Several amino acid residues proximal to the IL-2–IL-2Rα interaction interface were selected as candidate modification sites (Figure 1b). At each selected site, the corresponding codon in the IL-2 gene was substituted with an amber stop codon (TAG) to enable site-specific incorporation of the tetrazine-bearing noncanonical amino acid Tet v2.0,^39^ thereby facilitating efficient and selective PEG conjugation. TCO-PEG20K or TCO-GPLGLAGC-PEG20K was then conjugated to IL-2 via IEDDA reaction. The resulting TCO-GPLGLAGC-PEG20K-modified IL-2 variants were efficiently and completely cleaved by MMP-2 in vitro within 6 h at 37 °C (Figure 1c,d). Binding affinities of the PEGylated IL-2 variants and their corresponding cleaved products to IL-2Rα were quantified by biolayer interferometry (BLI) to facilitate high-throughput screening (Figure 1e and Table S2). Among the evaluated sites, K35 was selected for subsequent studies, as PEG conjugation at this position imposed strong steric shielding of IL-2Rα engagement while allowing near-complete recovery of binding affinity after cleavage, thereby generating a pronounced affinity contrast (9.57-fold) between the masked and unmasked states. Although PEGylation at K64 produced a larger activation fold-change following cleavage, the masked K64 variant retained higher residual activity prior to activation, indicating less stringent suppression in the caged state. Given our goal of minimizing unintended peripheral IL-2 signaling while preserving efficient protease-dependent activation, K35 provided a more favorable balance between activity suppression and conditional activation. In addition, the K35 variant exhibited higher expression yields during production and was therefore selected for further development. An uncleavable K35-PEGylated IL-2 variant was therefore generated as a control and termed chemically masked IL-2 (Cm-IL2) (Figure S2b,c). Although the linker constructs differed between Cm-IL2 and Cm-proIL2, both molecules exhibited comparable binding affinities to IL-2Rα (Figure S2c,d).

To further quantify the recovery of IL-2Rα binding following PEG removal, we measured binding affinities by surface plasmon resonance (SPR) before and after MMP-2 cleavage (Figure 1f). Cm-proIL2 exhibited a dissociation constant (*K*_D_) of 841 nM, representing a 26.7-fold reduction in affinity relative to wild-type IL-2 (*K*_D_ = 31.4 nM). In contrast, the cleaved Cm-proIL2 product, retaining the PLG remnant from PLGLAG linker cleavage, displayed a *K*_D_ of 81.7 nM, corresponding to an approximately 10-fold increase in affinity compared with uncleaved Cm-proIL2 and closely approaching that of wild-type IL-2, indicating near-complete functional recovery.

While PEGylation is often associated with broad steric effects on receptor engagement, the site-specific nature of our platform enables precise modulation of individual receptor interfaces. To directly assess whether PEG conjugation affected IL-2Rβγ engagement, we performed additional BLI analyses using Cm-IL2, Cm-proIL2, MMP-2-cleaved Cm-proIL2, and IL2-WT (Figure S3). All four proteins exhibited comparable binding affinities toward IL-2Rβγ, indicating that modification of the IL-2Rα interface by PEG does not substantially impair interaction with the IL-2Rβγ signaling receptor complex. These findings support the premise that site-specific PEGylation can selectively attenuate IL-2Rα binding while largely preserving IL-2Rβγ engagement, thereby enabling fine-tuning of receptor affinity and the establishment of receptor bias.

To assess the stability of the cleavable linker in circulation, we next evaluated the pharmacokinetic properties of Cm-IL2 and Cm-proIL2 in healthy C57BL/6J mice. A single intravenous dose of 0.5 mg kg^–1^ IL-2 conjugates was administered, and serum IL-2 concentrations were measured over time by ELISA (Figure 2a). Cm-IL2 exhibited a terminal half-life of 15.11 ± 0.13 h, whereas Cm-proIL2 displayed a comparable terminal half-life of 14.00 ± 0.37 h (Figure 2b). Importantly, no significant difference in area under the curve (AUC) was observed between Cm-IL2 (25,851.9 ± 1170.8 ng mL^–1^ h) and Cm-proIL2 (21,805.9 ± 2999.0 ng mL^–1^ h) (Figure 2c). These data demonstrate that, independent of tumor microenvironment-associated factors, Cm-proIL2 maintains systemic drug exposure comparable to that of Cm-IL2.

**Figure 2:**
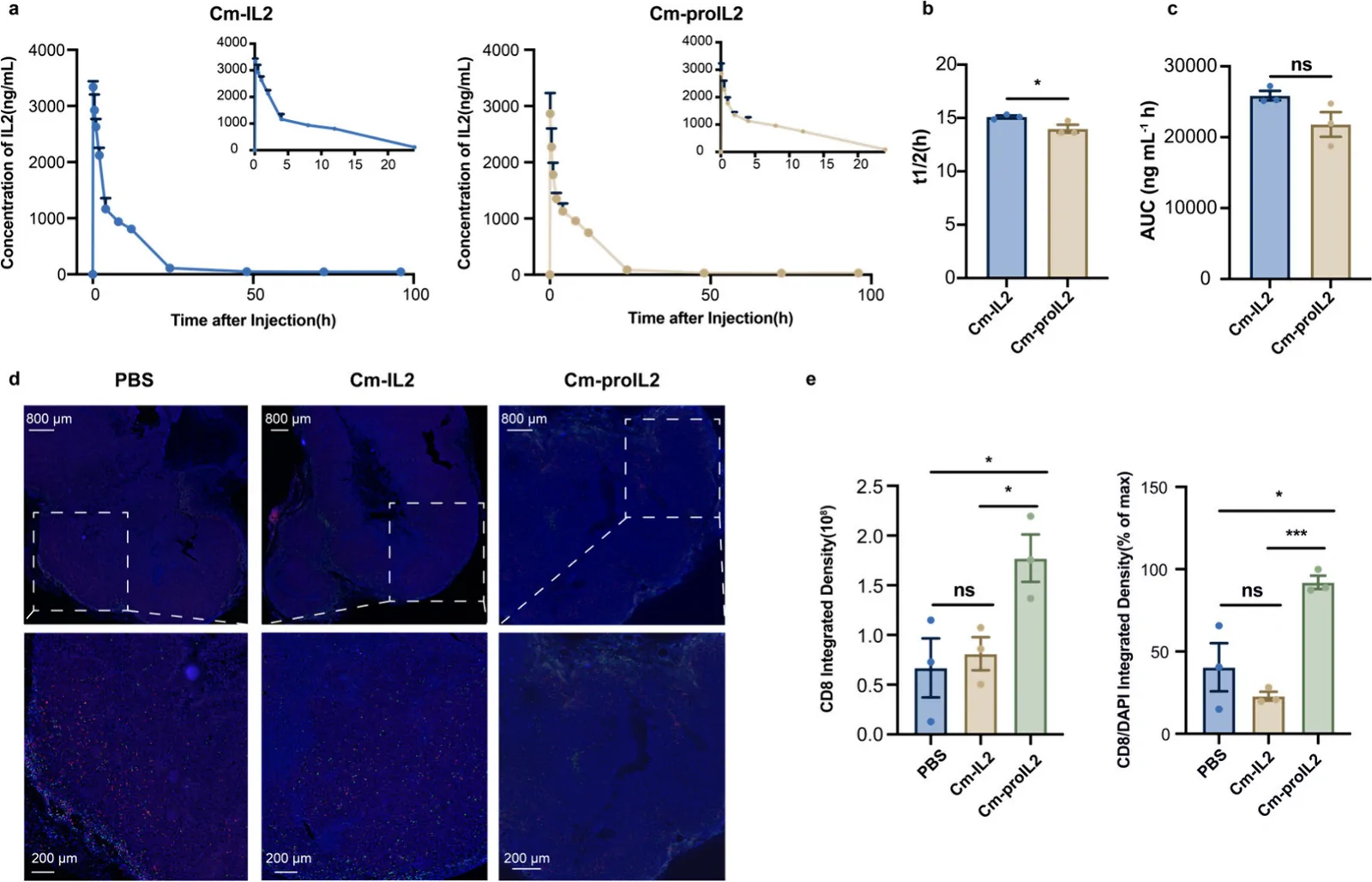
**Pharmacokinetic and lymphocyte penetration characterization of Cm-proIL2**. (a) Serum concentration–time curves of Cm-IL2 and Cm-proIL2 following intravenous administration. (b) Terminal half-life and (c) area under the curve (AUC) of Cm-IL2 and Cm-proIL2. *n* = 3 mice per group. (d) Representative immunofluorescence images showing intratumoral CD8^+^ T-cell infiltration. Nuclei were stained with DAPI (blue), CD3 with FITC (green), and CD8 with BV570 (red). (e) Semiquantitative analysis of fluorescence intensity from (d) using ImageJ, indicating enhanced lymphoid infiltration. Pharmacokinetic parameters were calculated using Phoenix WinNonlin 8.1. The data are presented as mean ± SEM. *P* values were determined by unpaired two-tailed *t* tests for the data in the bar graphs: *P < 0.05, **P < 0.01, ***P < 0.001.

Given the substantial reduction in molecular size following PEG cleavage, we hypothesized that Cm-proIL2 would promote enhanced intratumoral lymphocyte infiltration. To evaluate intratumoral T-cell distribution, tumor tissues were harvested from mice treated with Cm-proIL2, Cm-IL2, or PBS after three doses, with sacrifice occurring 2 days after the final injection. Immunofluorescence analysis of maximal tumor cross sections revealed increased CD8^+^ T-cell accumulation in tumors from Cm-proIL2-treated mice (Figure 2d). Semiquantitative analysis of CD8 integrated density or CD8/DAPI integrated density ratio further demonstrated a higher abundance of CD8^+^ T cells within deeper tumor regions compared with Cm-IL2 or PBS controls (Figure 2e). These results indicate that Cm-proIL2 induces markedly enhanced lymphoid infiltration within the tumors.

### Chemically Masked ProIL-2 Mediates Enhanced Antitumor Effect

To evaluate the antitumor efficacy of the TME-responsive chemically masked IL-2, MC38 tumor-bearing mice were intravenously administered 500 pmol of Cm-IL2 or Cm-proIL2 at 2 day intervals for a total of three doses (Figure 3a). Tumor volumes were measured longitudinally throughout the treatment and follow-up period (Figure 3b,c). Cm-proIL2 treatment led to a pronounced and sustained suppression of tumor growth compared with Cm-IL2, resulting in continuous tumor regression that was maintained for at least 2 weeks after the final administration without additional dosing.

**Figure 3:**
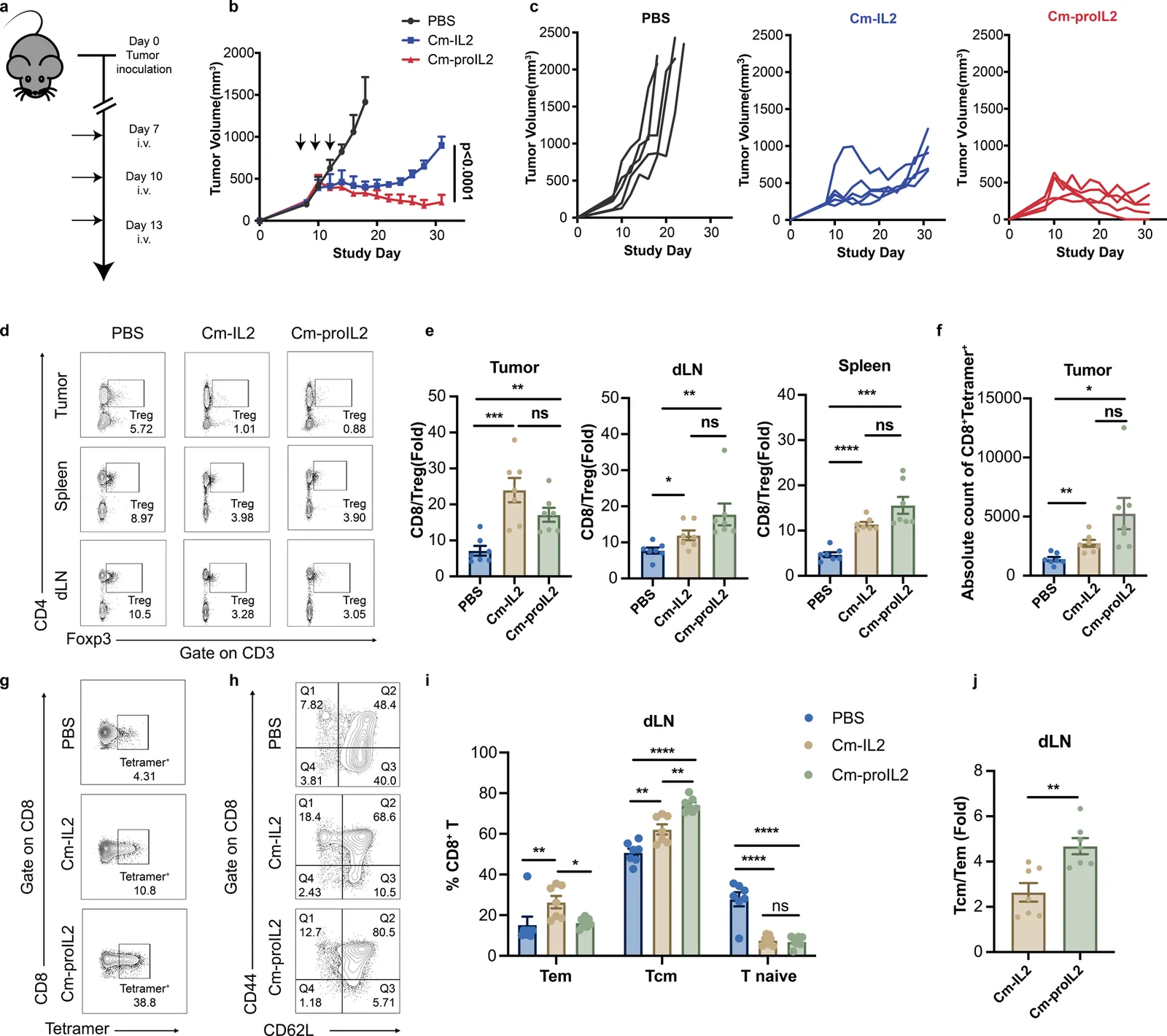
**Cm-proIL2-mediated robust antitumor efficacy.** (a) Schematic of the dosage regimen. Cm-IL2 or Cm-proIL2 was given at 2 day intervals for a total of three doses. (b) In vivo efficacy of Cm-IL2 and Cm-proIL2 in MC38 colorectal carcinoma. (c) Individual tumor growth curves of (b). *n* = 5 mice per group. (d) Representative dot plots showing Tregs in tumors, spleens, and tumor-draining lymph nodes (dLNs) of PBS-, Cm-IL2-, and Cm-proIL2-treated mice. (e) Bar graphs showing CD8^+^ T cell-to-Treg cell ratio of PBS-, Cm-IL2-, and Cm-proIL2-treated mice in tumors, dLNs, and spleens, respectively. *n* = 7 mice per group. (f) Bar graphs and (g) representative dot plots showing p15E-tetramer^+^ CD8^+^ T cells of PBS-, Cm-IL2-, and Cm-proIL2-treated mice in tumors *n* = 7 mice per group. (h) Representative dot plots showing CD8^+^ T cells characterized with CD44 and CD62L from dLNs. Effector memory T cells (Tems) were characterized as CD62L^–^CD44^+^ (Q1); central memory T cells (Tcms) were characterized as CD62L^+^CD44^+^ (Q2); and naive T cells were characterized as CD62L^+^CD44^–^ (Q3). (i) Bar graphs showing frequencies of Tem, Tcm, and naive T cells within CD8^+^ T cells of PBS-, Cm-IL2-, and Cm-proIL2-treated mice in dLNs. (j) Bar graphs showing Tcm-to-Tem cell ratio of Cm-IL2- and Cm-proIL2-treated mice in dLNs. *n* = 7 mice per group. The data are presented as mean ± SEM. *P* values were determined by two-way ANOVA for tumor growth data and by unpaired two-tailed *t* tests for the data in the bar graphs: *P < 0.05, **P < 0.01, ***P < 0.001, ****P < 0.0001.

To gain insight into the immunological correlates associated with the enhanced antitumor efficacy of Cm-proIL2, tumors and peripheral immune compartments, including tumor-draining lymph nodes (dLNs) and spleens, were collected from mice treated with Cm-IL2, Cm-proIL2 or PBS for immune subset analysis (Figure S4). Mice received three doses of treatment and were euthanized 3 days after the final injection. Despite the recovery of IL-2Rα binding following MMP-mediated cleavage of Cm-proIL2 within the tumor microenvironment, no significant differences in Treg frequencies were observed between the Cm-IL2 and Cm-proIL2 groups across tumors, dLNs or spleens (Figure 3d,e). Consistent with this observation, both IL-2 formulations resulted in an increased CD8^+^ T cell-to-Treg ratio relative to PBS-treated controls. Analysis of antigen-specific responses revealed that MC38-derived p15E tetramer^+^ CD8^+^ T cells were similarly expanded in both Cm-IL2- and Cm-proIL2-treated mice compared with PBS controls, with no significant difference in their overall abundance between the two IL-2 formulations (Figure 3f,g). These results indicated that the superior antitumor efficacy of Cm-proIL2 is not associated with a further increase in the total number of tumor-specific CD8^+^ T cells.

To further explore cellular features associated with the enhanced durability of Cm-proIL2-mediated antitumor responses beyond lymphoid infiltration, memory T cell subsets were analyzed in tumors, dLNs and spleens (Figure 3h). No substantial differences in memory T cell composition were observed in tumors or spleens between treatment groups (Figure S5), while both IL-2 formulations led to a pronounced reduction in naïve T cells compared with PBS-treated controls. In contrast, within dLNs, Cm-proIL2 treatment was associated with a higher proportion of central memory T cells (Tcm), whereas Cm-IL2 preferentially promoted effector memory T cells (Tem) (Figure 3i). Consequently, Cm-proIL2 resulted in an increased Tcm-to-Tem ratio in dLNs, a pattern consistent with its more durable antitumor efficacy (Figure 3j).

### Chemically Masked ProIL-2 Improves CD8^+^ T Cell Functionality Within the Tumor Microenvironment

To further characterize immune cell states within the tumor microenvironment associated with Cm-proIL2 treatment, we performed single-cell RNA sequencing (scRNA-seq) analysis of tumor-infiltrating lymphocytes (TILs) from MC38 tumor-bearing mice treated with PBS, Cm-IL2, or Cm-proIL2, with a focus on T cell population distributions (Figures 4a and Figure S6). Cell type annotation revealed that Cm-proIL2 treatment was associated with a reduced proportion of regulatory T cells (Tregs; 5.25%) compared with Cm-IL2 (7.69%) and PBS (20.3%), accompanied by increased frequencies of proliferating T cells (7.25% for Cm-proIL2, 5.01% for Cm-IL2, and 6.93% for PBS) and memory T cells (Figure 4b).

**Figure 4:**
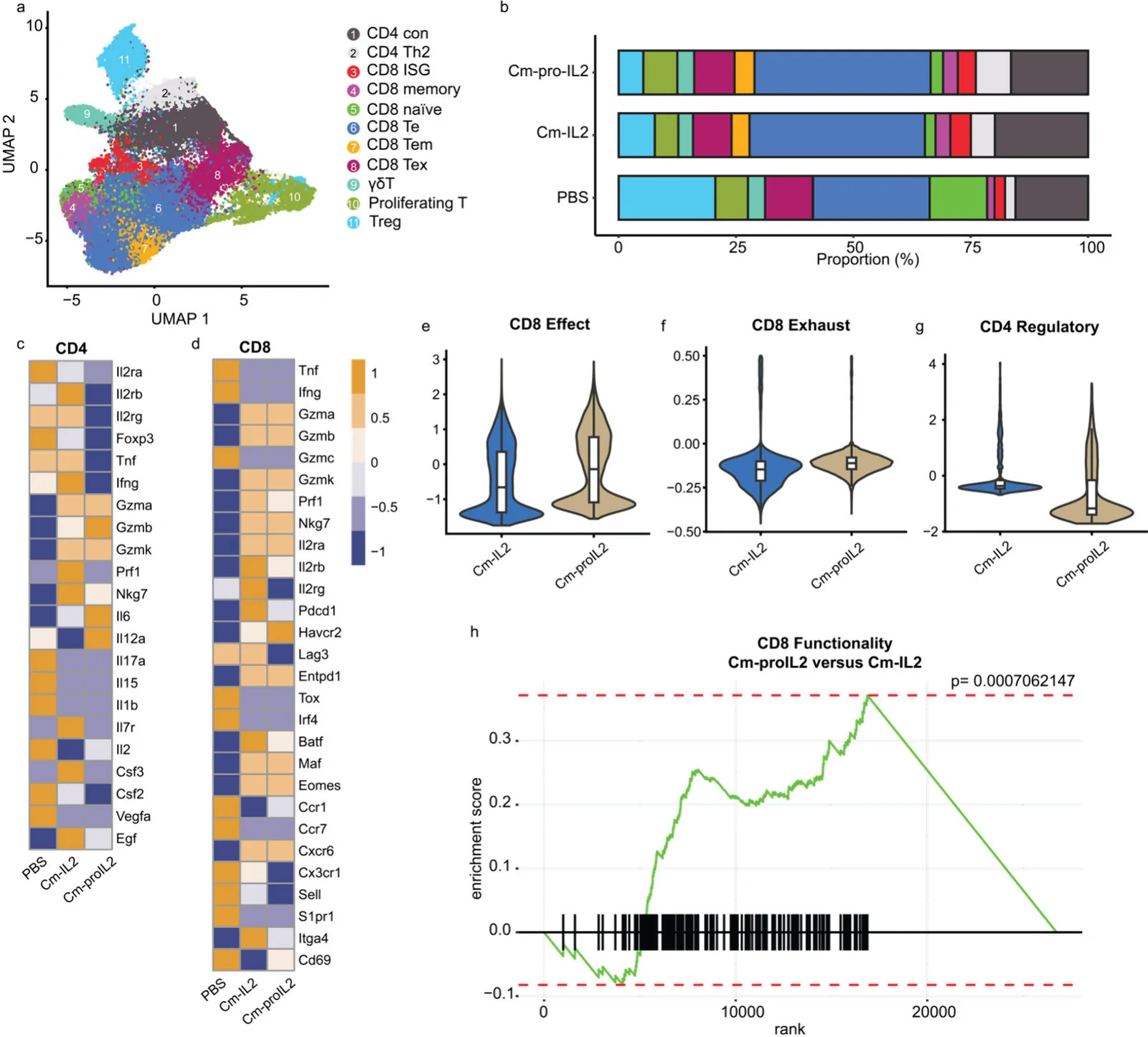
**Cm-proIL2 is associated with enhanced T cell activation states within the tumor microenvironment**. (a) UMAP visualization of major CD3^+^ T cell clusters identified from tumors of mice treated with PBS, Cm-IL2, or Cm-proIL2. (b) Relative proportions of T cell subsets corresponding to the clusters shown in (a). Cluster colors are consistent between panels. (c, d) Heatmap visualizations of selected functional gene expression in (c) CD4^+^ T cells and (d) CD8^+^ T cells across treatment groups. (e, f) Violin plots comparing enrichment scores of (e) effector-associated and (f) exhaustion-associated gene signatures in CD8^+^ T cells. (g) Violin plot showing enrichment of a regulatory-associated gene signature in CD4^+^ T cells. (h) Gene set enrichment analysis (GSEA) comparing Cm-proIL2- versus Cm-IL2-treated CD8^+^ T cells. GSEA was performed using a two-sided permutation test.

Consistent with these population-level changes, gene expression analysis showed that both Cm-IL2 and Cm-proIL2 treatments were associated with reduced expression of regulatory-associated genes, including *Foxp3*, in CD4^+^ T cells (Figure 4c), as well as increased expression of effector-associated genes, including *Gzma*, *Gzmb*, *Gzmk*, *Prf1*, and *Nkg7*, in CD8^+^ T cells relative to PBS controls (Figure 4d). Notably, no significant differences in the expression of these genes were observed between the Cm-proIL2 and the Cm-IL2 groups.

To further characterize CD8^+^ T cell functional states in response to Cm-IL2 versus Cm-proIL2 treatment, we defined two gene expression signatures^15,40,41^ reflecting effector-associated and exhaustion-associated transcriptional programs. The effector-associated signature, comprising *Gzmk*, *Gzmb*, *Ifng*, *Prf1*, *Ifit3*, *Ifit1*, *Havcr2*, *Pdcd1*, and *Lag3*, was more highly enriched in CD8^+^ T cells from the Cm-proIL2-treated group compared with the Cm-IL2 group (Figure 4e). In parallel, an exhaustion-associated signature defined by *Lag3*, *Tcf7*, *Havcr2*, and *Pdcd1* was also elevated in CD8^+^ T cells following Cm-proIL2 treatment (Figure 4f). Notably, increased expression of exhaustion-associated genes occurred in parallel with enhanced expression of cytotoxic and effector molecules, suggesting that Cm-proIL2 promotes a highly activated tumor-infiltrating CD8^+^ T-cell state accompanied by the adaptive upregulation of inhibitory receptors rather than isolated functional impairment. This observation may have implications for the durability of cytokine monotherapy and provides additional rationale for PD-1-targeted Cm-proIL2.

Given the role of Tregs in modulating CD8^+^ T cell responses, transcriptional features of Tregs were also examined. A regulatory-associated signature defined by *Il10*, *Tgfb1*, *Il6*, and *Foxp3* was significantly reduced in the Cm-proIL2-treated group compared with Cm-IL2 (Figure 4g). Together, these transcriptional profiles suggest that Cm-proIL2 treatment is associated with a more functionally activated T cell landscape within the tumor microenvironment. These findings indicate that Cm-proIL2 is associated with enhanced activation of CD8^+^ T cells, accompanied by increased expression of inhibitory or differentiation-associated markers. Consistently, gene set enrichment analysis (GSEA) revealed enrichment of a CD8^+^ T cell functional gene set in the Cm-proIL2 group (Figure 4h).

### PD-1 Targeted Chemically Masked IL-2 Eradicates Mouse Colorectal Carcinoma

Previous studies have shown that IL-2 can synergize with anti-PD-1 therapy to enhance antitumor efficacy.^15,21,42^ In our scRNA-seq analysis, Cm-proIL2 treatment was associated with an increased exhaustion state of CD8^+^ T cells (Figure 4f). Based on this observation and to further enhance antitumor efficacy, we designed PD-1-targeted IL-2 constructs by fusing the PD-1-binding nanobody Nb11,^43–45^ which is cross-reactive between human and murine PD-1, to the N-terminus of Cm-IL2 and Cm-proIL2. The resulting PD1-Cm-IL2 and PD1-Cm-proIL2 fusion proteins enable PD-1-directed cis-presentation of IL-2 to T cells, thereby enriching IL-2 signaling within the tumor immune microenvironment while limiting peripheral exposure (Figure 5a,b). The binding profiles of PD1-Cm-IL2 and PD1-Cm-proIL2 toward PD-1, IL-2Rα, and IL-2Rβγ were evaluated (Figure S7), demonstrating that the PD-1 nanobody retains its target-binding capability while remaining functionally compatible with the engineered IL-2 cytokine module and its receptor interactions.

**Figure 5:**
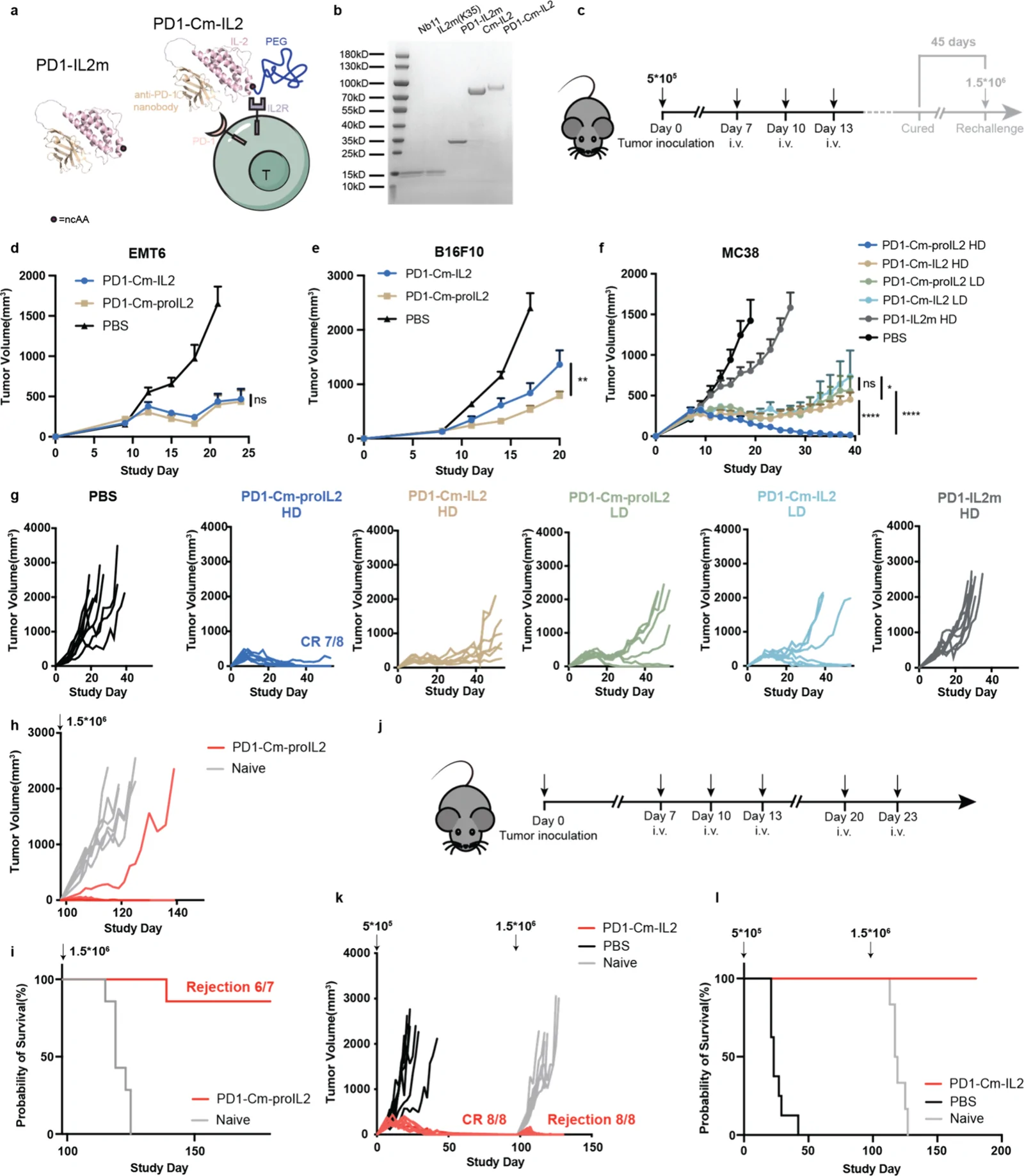
**In vivo efficacy of PD1-Cm-IL2 and PD1-Cm-proIL2**. (a) Schematic of cis presentation of Cm-IL2 and Cm-proIL2 via PD-1 targeting. The predicted structure of Nb11-IL2 was generated using AlphaFold 3. (b) SDS-PAGE verification of different IL-2-based scaffolds. (c) Schematic of dosage regimen. PD1-Cm-IL2 or PD1-Cm-proIL2 was given at 2 day intervals for a total of three doses. (d, e) Tumor growth curves in (d) EMT6 and (e) B16F10 tumor models. 0.75 mg per kg (body weight) was intravenously injected. *n* = 8 mice per group. (f) Tumor growth curves and (g) individual tumor growth in MC38 tumor model of mice treated with PBS, 250 pmol (low-dose, LD) or 500 pmol (high-dose, HD) IL-2 PEG conjugates or 500 pmol PD1-IL2m (non-PEGylated). All treatments were intravenously injected at a 2 day interval for a total of three doses starting from Day 7 after tumor inoculation when tumor volumes exceeded 200 mm^3^. *n* = 8 mice per group. (h, i) Rechallenge study of cured mice from (f): (h) tumor growth curves and (i) survival curves of the rechallenge study. Surviving mice were rechallenged with 3 times the original dose of MC38 cells. (j) Schematic of dosage regimen for PD1-Cm-IL2. In vivo efficacy of PD1-Cm-IL2 in MC38 tumor model for mice given another booster treatment period. (k) Tumor growth curves and (l) survival curves for the therapeutic efficacy study in (j), including rechallenge study. Mice were sacrificed when the tumor volume reached over 2000 mm^3^ or the body weight loss was over 20%. The data are presented as mean ± SEM. *P* values were determined by two-way ANOVA for tumor growth data: *P < 0.05, **P < 0.01, ***P < 0.001, ****P < 0.0001.

To evaluate the in vivo therapeutic efficacy of the PD-1-targeted IL-2 derivatives, we first tested these constructs at a suboptimal dose in murine EMT6 mammary adenocarcinoma and B16F10 melanoma models. Tumor-bearing mice were intravenously administered PD1-Cm-IL2 or PD1-Cm-proIL2 at 0.75 mg kg^–1^ at 2 day intervals for a total of three doses, with PBS-treated mice serving as controls (Figure 5c). In the EMT6 model, both PD1-Cm-IL2 and PD1-Cm-proIL2 elicited potent antitumor activity, with no significant difference observed between the two treatments (Figure 5d). In contrast, in the B16F10 model, PD1-Cm-proIL2 exhibited a more pronounced antitumor effect compared with PD1-Cm-IL2 (Figure 5e). The model-dependent benefit of PD1-Cm-proIL2 may reflect differences in intratumoral delivery constraints and protease activity between EMT6 and B16F10 tumors, which could modulate PEG removal and cytokine distribution within the TME.

To further improve therapeutic efficacy, we next optimized dosing and evaluated the PD-1-targeted IL-2 constructs in the MC38 colorectal tumor model, which exhibits higher immunogenicity and MMP expression (Figure S1). MC38-bearing C57BL/6J mice were intravenously administered PD1-Cm-IL2 or PD1-Cm-proIL2 at 250 pmol (∼7.5 μg) or 500 pmol (∼15 μg) every 2 days for a total of three doses, and tumor growth was monitored over time (Figure 5f,g). As an additional comparator, a non-PEGylated construct (PD1-IL2m), in which the IL-2 mutant K35Tet v2.0 was fused to Nb11, was included.

Dose escalation resulted in enhanced antitumor activity, with the high-dose regimen (HD, 500 pmol) providing more durable tumor growth control than the low-dose regimen (LD, 250 pmol). Under the low-dose condition, no clear difference was observed between PD1-Cm-IL2 and PD1-Cm-proIL2. In contrast, at the high dose, PD1-Cm-proIL2 exhibited markedly stronger antitumor efficacy, achieving complete tumor regression in seven out of eight treated mice (Figure 5g). Mice that achieved complete tumor regression following PD1-Cm-proIL2 treatment were rechallenged with MC38 cells after a treatment-free interval of at least 45 days (Figure 5c). The majority of rechallenged mice (six out of seven) rejected tumor growth, indicating durable tumor protection (Figure 5h,i).

Unlike PD1-Cm-proIL2, PD1-Cm-IL2 did not induce complete tumor regression under the standard three-dose regimen. We therefore evaluated whether extending the dosing schedule could further improve therapeutic outcomes and define the therapeutic ceiling of PD1-Cm-IL2. To this end, two additional doses of PD1-Cm-IL2 were administered following the initial treatment course (Figure 5j). Under this extended regimen, complete tumor regression was achieved. To assess the durability of these responses, cured mice were rechallenged with MC38 cells using the same protocol as that in the PD1-Cm-proIL2 rechallenge experiment. All rechallenged mice (eight out of eight) rejected tumor growth (Figure 5k,l). Together, these results indicate that while PD1-Cm-IL2 can achieve complete tumor control with additional dosing, PD1-Cm-proIL2 reaches comparable therapeutic end points under a shorter dosing regimen.

### Immune Reprogramming and Systemic Safety of PD-1-Targeted Chemically Masked ProIL-2

To assess how PD1-Cm-IL2 and PD1-Cm-proIL2 remodel the TME, we first examined their intratumoral distribution by performing an immunofluorescence analysis on the largest cross-sectional regions of tumors collected after three doses of treatment with PD1-Cm-IL2, PD1-Cm-proIL2, or PBS. Representative images revealed that PD1-Cm-proIL2 treatment resulted in a more spatially extensive distribution of CD8^+^ T cells within tumors, with increased accumulation in the tumor core compared with PD1-Cm-IL2-treated controls (Figure 6a). Notably, tumor sections from the PD1-Cm-proIL2 group contained numerous void-like structures, which may indicate erosion of tumor tissue from the interior.

**Figure 6:**
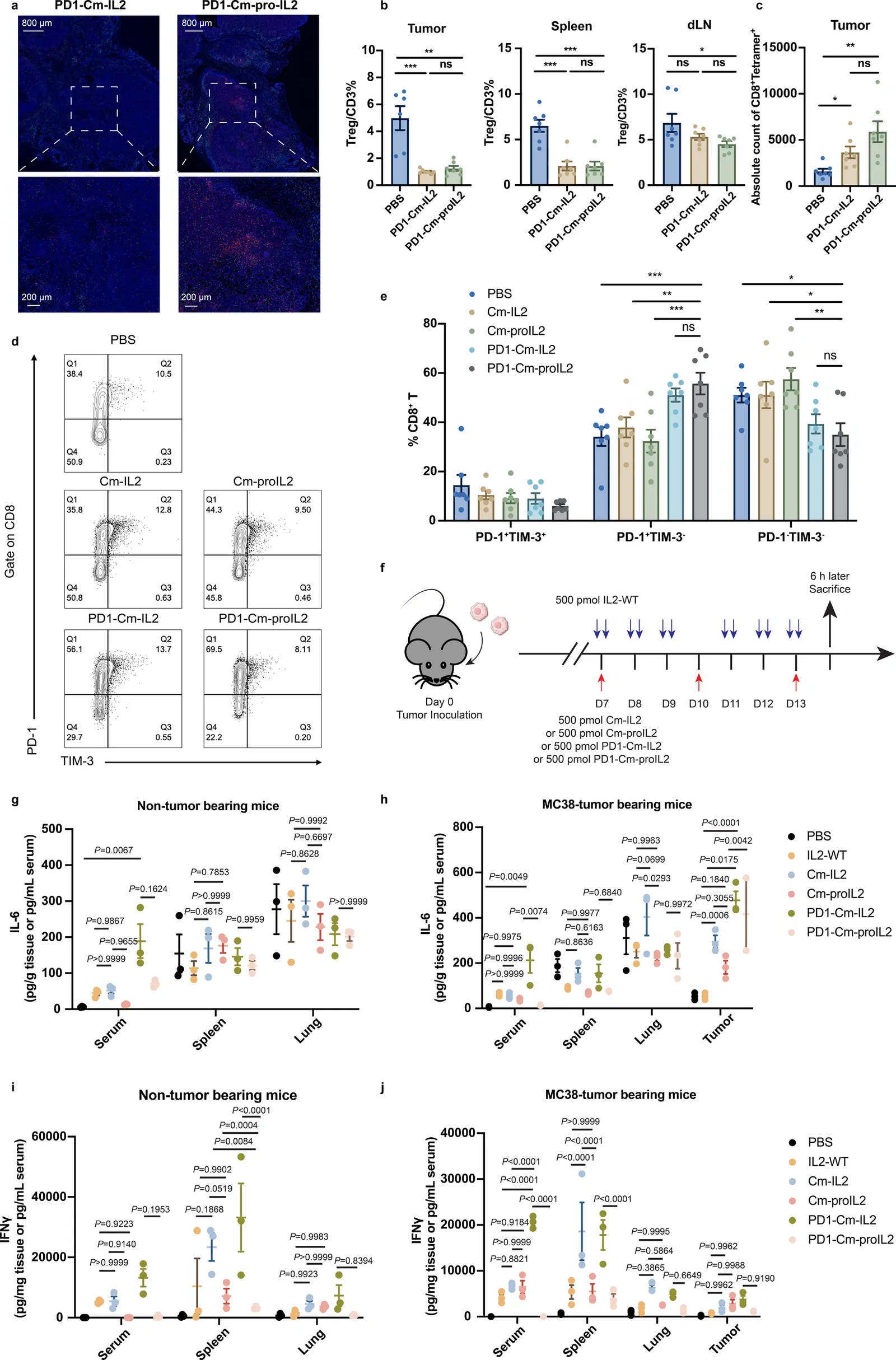
**Immune profiling and systemic safety evaluation of PD1-Cm-proIL2.** (a) Representative immunofluorescence images showing CD8^+^ T cell infiltration in the maximal cross section of tumors. Nuclei were labeled with DAPI (blue), CD3 were labeled with FITC (green) and CD8 were labeled with BV570­(red). (b, c) Bar graphs showing (b) frequencies of Treg cells within CD3^+^ T cells of PBS-, PD1-Cm-IL2-, and PD1-Cm-proIL2-treated mice in tumors, dLNs, and spleens and (c) p15E-tetramer^+^ CD8^+^ T cells in tumors, respectively. (d) Representative dot plots showing CD8^+^ T cells characterized with PD-1 and TIM-3 from tumors. (e), Bar graphs showing frequencies of PD-1^–^TIM-3^+^, PD-1^+^TIM-3^–^, and PD-1^–^TIM-3^–^ cells of PBS-, Cm-IL2-, Cm-proIL2-, PD1-Cm-IL2-, and PD1-Cm-proIL2-treated mice in tumors. (f) Schematic of the safety study design. (g, h) IL-6 levels in serum and the indicated tissues in (g) non-tumor-bearing and (h) MC38 tumor-bearing mice treated with the indicated IL-2 constructs. (i, j) IFNγ levels in serum and the indicated tissues in (i) non-tumor-bearing and (j) MC38 tumor-bearing mice treated with the indicated IL-2 constructs. The data are presented as mean ± SEM. *P* values were determined by unpaired two-tailed *t* tests for the data in the bar graphs: *P < 0.05, **P < 0.01, ***P < 0.001, ****P < 0.0001.

To further characterize immune remodeling within the tumor microenvironment, we performed flow cytometric analysis of T cell subsets following treatment (Figure S8). MC38 tumor-bearing mice were randomized to receive PBS, PD1-Cm-IL2, or PD1-Cm-proIL2 for three doses administered at 2 day intervals, and spleens, dLNs, and tumors were collected 72 h after the final dose. Both PD1-Cm-IL2 and PD1-Cm-proIL2 treatments resulted in reduced frequencies of Tregs not only within tumors but also across peripheral immune compartments, including spleens and dLNs (Figure 6b). In parallel, both treatments increased the abundance of MC38 antigen–specific p15E-tetramer^+^ CD8^+^ T cells relative to PBS controls; however, no significant difference was observed between the two treatment groups (Figure 6c).

To explore the potential synergistic effects of PD-1 blockade within the TME, we assessed T cell exhaustion by staining tumor-infiltrating lymphocytes with anti-PD-1 and TIM-3 antibodies (Figure 6d). Fusion of Cm-IL2 or Cm-proIL2 to the anti-PD-1 nanobody Nb11 promoted expansion of PD-1^+^TIM-3^–^ T cells while reducing the population of nonexhausted, PD-1^–^TIM-3^–^ T cells (Figure 6e). No substantial differences were detected between PD1-Cm-IL2 and PD1-Cm-proIL2 across these T cell subsets. Together, these findings indicate that the superior antitumor efficacy of PD1-Cm-proIL2 is primarily associated with enhanced intratumoral penetration and the resulting increase in CD8^+^ T-cell infiltration rather than differences in the overall abundance or phenotypic composition of antigen-specific T cells.

Given the systemic toxicities, including anemia, reported in early clinical studies of IL-2-based therapies, we first assessed the safety of PD1-Cm-IL2 and PD1-Cm-proIL2 in a single-dose setting (Figure S9). MC38 tumor-bearing mice received a single intravenous dose (500 pmol), with blood collected 5 days postinjection and major organs harvested at day 7 for histological analysis. Complete blood count (CBC) analysis showed increased circulating leukocyte populations, including lymphocytes, neutrophils, monocytes, eosinophils, and basophils, in mice treated with either fusion protein, consistent with systemic immune activation. In contrast, red blood cell indices (HGB, RBC, and HCT) remained comparable across groups, indicating no evidence of treatment-induced anemia. Serum creatine kinase (CK) levels were similar among all groups, and lactate dehydrogenase (LDH) levels were reduced in both treatment groups compared with PBS, likely reflecting reduced tumor burden rather than tissue injury. Consistently, histopathological examination of major organs, including heart, liver, spleen, lung, and kidney, revealed no detectable treatment-related abnormalities. No evidence of pulmonary edema was observed in either cytokine-treated group.

We next performed a systematic safety evaluation using a treatment regimen consistent with that employed in the efficacy studies (Figure 6f). Mice were euthanized 6 h after completion of one treatment cycle, corresponding to approximately half of the circulating half-life of the constructs, and samples were collected for assessment of inflammatory cytokines, organ toxicity, and pulmonary edema. Native IL-2 (IL2-WT) was included as a control; however, given its extremely short half-life (∼5 min), even twice-daily administration for 3 consecutive days could not fully recapitulate clinically relevant high-dose infusion regimens.

At study end, IL2-WT-treated mice exhibited the largest tumor burden among all groups, consistent with its limited in vivo exposure (Figure S10a). Assessment of pulmonary toxicity revealed increased lung weights in mice treated with PD-1-targeted IL-2 constructs, whereas no significant differences were observed among IL2-WT, Cm-IL2, and Cm-proIL2 (Figure S10b). Inflammatory cytokine analysis (Figure 6g–j) showed that Cm-IL2 induced higher splenic IFNγ levels than IL2-WT and Cm-proIL2, while PD1-Cm-IL2 further increased IFNγ levels in both blood and spleen, indicating enhanced systemic immune activation associated with PD-1-directed targeting. Notably, incorporation of the chemically masked prodrug design markedly attenuated cytokine induction while preserving antitumor efficacy.

Collectively, these findings indicate that PD-1-directed cytokine targeting enhances immune activation but is accompanied by increased systemic inflammatory responses, whereas chemical masking substantially improves the safety profiles of both conventional and PD-1-targeted IL-2 constructs.

## Discussion

Interleukin-2 (IL-2) holds substantial promise for cancer immunotherapy; however, its paradoxical activation of both effector T cells and regulatory T cells has long constrained its clinical utility. While reducing IL-2Rα affinity successfully limits Treg activation, optimal expansion and durability of antigen-specific effector T cells still rely heavily on IL-2Rα interaction. Activated effector T cells depend on upregulated IL-2Rα to assemble high-affinity receptor complexes, creating a feedback loop essential for capturing limiting concentrations of IL-2 within the tumor microenvironment.^15,42^ Preclinical and clinical experiences with heavily receptor-biased variants suggest that an overattenuation of this pathway can limit the selective expansion of these tumor-reactive T cells. These findings highlight that retaining a baseline level of IL-2Rα engagement may be necessary to sustain potent, antigen-targeted antitumor responses. In this work, we therefore adopted a strategy that selectively shields IL-2Rα engagement in peripheral tissues to reduce off-target Treg activation and systemic cytokine consumption, while enabling full restoration of IL-2 activity within the tumor microenvironment. Through site-specific PEG conjugation, Cm-proIL2 transiently masks IL-2Rα binding in circulation, thereby limiting peripheral immune suppression without permanently attenuating cytokine function. Importantly, PEG does not intrinsically interact with IL-2, allowing complete recovery of receptor binding following proteolytic cleavage in the tumor microenvironment. This design preserves the intrinsic potency of IL-2 while improving the balance between systemic safety and intratumoral immune activation. The combined effects of reduced peripheral cytokine consumption and restored local IL-2 signaling collectively contribute to a broadened therapeutic window.

Beyond modulation of receptor engagement, a key advantage of the chemically masked procytokine strategy lies in its impact on intratumoral immune distribution. Immune subset analyses revealed no significant differences in the overall abundance of CD8^+^ T cells or tumor antigen-specific T cells between Cm-proIL2 and the noncleavable Cm-IL2, regardless of PD-1 targeting. In contrast, immunofluorescence analyses consistently demonstrated markedly enhanced lymphocyte infiltration in tumors treated with Cm-proIL2. These data indicate that improved spatial distribution and penetration of lymphocytes, rather than increased lymphocyte quantity, underlie the superior antitumor efficacy of the chemically masked proIL-2.

An additional conceptual advantage of this strategy is the use of polyethylene glycol as a chemically orthogonal and tunable masking module. The PEG chain length can be readily adjusted to modulate pharmacokinetic properties, and the same masking principle may be adaptable to other cytokines provided that suitable engineering sites can be identified without compromising protein function. These features suggest the potential broader applicability of chemically masked procytokines beyond IL-2, although the scope of this approach remains to be established for individual cytokine systems.

In summary, we developed a chemically masked IL-2 prodrug and a PD-1-targeted IL-2 prodrug bispecific, among which PD1-Cm-proIL2 elicited potent antitumor activity and durable protective immunity in multiple solid tumor models. In this system, PEG conjugation fulfills a trifunctional role by extending systemic half-life, selectively shielding IL-2Rα engagement in circulation while enabling restoration of receptor binding within the tumor microenvironment and reducing the effective molecular size to facilitate tumor penetration and lymphocyte infiltration. This modular, tumor microenvironment-responsive immunocytokine platform provides a potential solution to the long-standing challenge of balancing cytokine efficacy and systemic safety and highlights the utility of chemically masked cytokine engineering for improving the therapeutic index of IL-2-based immunotherapy.

## Methods

### Ethical Statement

All animal experiments were conducted following approval by the Animal Ethics Committee of Peking University (Beijing, China) under the approval number LA2022397. The study adhered to the maximum tumor volume limit of 3.35 cm^3^ set by the Institutional Animal Care and Use Committee (IACUC) of Peking University, and this limit was not exceeded during the course of the study.

### General Information

All chemicals and reagents utilized in this study were obtained from established commercial suppliers. The DH5α strain was employed for plasmid DNA cloning, while the transB­(DE3) strain was used for protein expression. Polymerase Chain Reaction (PCR) was performed using KOD One (TOYOBO) polymerase. DNA fragments were purified with the DNA Clean-Up Kit (Vazyme), and plasmid preparations were carried out using the GeneJET Plasmid Miniprep Kit (Thermo). Protein purification procedures involved Ni-NTA (Promega), Capto SP ImpRes (Cytiva), and Superdex 75 (Cytiva) chromatography columns. Recombinant receptors were sourced from SinoBiological. The synthesis of the tetrazine-functionalized unnatural amino acid Tet v2.0 was conducted as described in prior studies.^39^ TCO-mPEG20K was purchased from Confluore Life Science Accelerator. The purity of the proteins and their Pegylated forms was verified by SDS-PAGE (Meilunbio), followed by Coomassie blue staining.

### Cell Lines and Animals

All cell lines used in this study were acquired from commercial vendors. MC38 cells (Pricella) were cultured in DMEM (Macgene) medium with 10% FBS and 1% Pen/Strep. B16F10 cells and EMT6 (Pricella) were cultured in RPMI 1640 (Macgene) medium with 10% FBS and 1% Pen/Strep. All cell cultures were maintained at 37 °C in a 5% CO_2_ atmosphere.

Six- to eight-week-old female C57BL/6J mice were purchased from the Department of Laboratory Animal Science at Peking University Health Science Center. The animals were kept in a controlled environment with a temperature range of 20–25 °C, humidity levels between 40% and 60%, and a 12 h light/dark cycle.

### Production of IL2 Mutants and Their PEG Conjugates

The sequence of anti-PD1 nanobody Nb11 was derived from patent WO2024083988A1, codon-optimized, and synthesized by General Biol. The resulting DNA fragment was cloned into the N-terminus of IL2 (K35TAG) via overlap PCR.

The plasmids carrying IL2K35TAG and Nb11-IL2K35TAG were cotransformed with pULTRA-aaRS, which includes an orthogonal *Mj*tRNA/Tet v2.0-specific aminoacyl-tRNA synthetase pair, into *Escherichia coli* strain TransB­(DE3). Expression of both IL2K35Tet v2.0 and Nb11-IL2K35Tet v2.0 was carried out at 25 °C for 12–16 h. Upon reaching an OD_600_ of 0.6–0.8, 0.6 mM IPTG and 1 mM Tet v2.0 were added to the culture medium. Afterward, the cells were harvested by centrifugation (5000 rpm for 30 min) and resuspended in binding buffer (20 mM Tris-HCl, pH 8.0, 500 mM NaCl, 10 mM imidazole) containing 1% TieChui *E. coli* lysis buffer (ACE). The supernatant was obtained by centrifugation (12,000 rpm, 30 min at 4 °C) and purified using Ni-NTA resin. The crude product was then further purified using Capto SP ImpRes, which was equilibrated in 20 mM sodium acetate (pH 4.5) containing 100 mM NaCl. Elution was carried out using a linear gradient from 0% to 100% over 10 column volumes of elution buffer (20 mM sodium acetate, pH 4.5, 1 M NaCl). The main elution peak was collected and concentrated for further analysis.

For site-specific PEG conjugation, 30 μM purified IL2K35Tet v2.0 and Nb11-IL2K35Tet v2.0 reacted with 150 μM TCO-PEG or TCO-GPLGLAGC-PEG at 25 °C overnight, the resulting mixture was purified with Superdex75. Endotoxin levels of the purified protein constructs were determined using a commercial endotoxin detection assay (GenScript, #L01055-20). All preparations contained 0.006–0.012 EU per mouse dose, well below the accepted daily endotoxin limit for experimental mice (0.1 EU per 20 g body weight).

### In Vitro MMP Cleavage

MMP-2 (SinoBiological) was activated following the protocol using TCNB buffer (G-CLONE). The resulting activated MMP-2 (10–20 μg/mL) was then diluted 10-fold into 20 μM Cm-proIL2 and incubated at 37 °C for 6–8 h. Cm-proIL2 could be cleaved completely after the reaction.

### Binding Affinity Evaluation

Binding affinity with CD25 of Cm-proIL2, Cm-proIL2 (PLG), and wild-type IL-2 was evaluated by SPR using a Biacore 8K+ (GE Healthcare). His-tagged human CD25 was immobilized on a CM5 sensor chip via an amine coupling. A 2-fold dilution series of three constructions (ranging from 15.62 to 2 000 nM) was tested in a nine-point titration, respectively. The dissociation of the interactions was monitored for 120 s. Following each measurement, the biosensors were regenerated by injecting glycine-HCl (pH 2.5, Cytiva) for 5 s, followed by PBS for 5 s, with this process repeated twice. The resulting data were analyzed using Biacore 8K Evaluation Software, and the binding kinetics, including *K*_D_ values, were determined by fitting the data to a 1:1 global fit model.

### Immunofluorescence Staining and Image Analysis of Lymphocyte Infiltration

Cm-IL2, Cm-proIL2, PD1-Cm-IL2, PD1-Cm-proIL2 (500 pmol), or PBS was injected intravenously into MC38 tumor-bearing C57BL/6J mice at 2 day intervals, with a total of three doses. Two days after the treatment period, the mice were sacrificed, and the tumors were isolated and fixed with 4% paraformaldehyde (PFA). Slides were stained with anti-mouse CD3 and CD8 antibodies and imaged with Vectra Polaris. The fluorescent intensity was analyzed with ImageJ.

### Pharmacokinetic Analysis of Cm-IL2 and Cm-proIL2

Female C57BL/6J mice were bled via the retro-orbital sinus 1 day before administration (0 h). Mice received intravenous IL-2 or its variants (0.5 mg/kg), and blood samples (50–100 μL) were collected at 0.5, 2, 8, 24, 48, 72, and 96 h postinjection into anticoagulant tubes. Serum was isolated by centrifugation (300*g*, 5 min, twice), snap-frozen in liquid nitrogen, and stored at −80 °C.

For ELISA, 96-well high-binding plates were coated overnight at 4 °C with 50 ng/well anti-His antibody (TransGene) in pH 9.6 coating buffer. Plates were washed four times with TBST and blocked with 5% BSA in TBST for 30 min at room temperature. Serum samples were diluted in 5% BSA/TBST and incubated for 2 h at room temperature. IL-2 or PEGylated IL-2 standards were prepared individually to account for PEG-induced binding differences. After washing, plates were incubated with 1 μg/mL HRP-conjugated anti-IL-2 antibody (SinoBiological) in 3% BSA/TBST for 1 h at room temperature. Following four washes, TMB substrate was added and incubated at 37 °C for 10–30 min. Reactions were stopped with TMB stop solution, and absorbance at 450 nm was measured using a plate reader.

### In Vivo Efficacy in Mice

To evaluate antitumor efficacy, 5 × 10^5^ B16F10 or EMT6 cells were injected subcutaneously into the right flank of mice in 100 μL PBS. Once the tumor volume reached approximately 200 mm^3^, the tumor-bearing mice were randomly assigned to treatment groups. 0.75 mg per kg (body weight) PD1-Cm-IL2 or PD1-Cm-proIL2 was injected intravenously at 2 day intervals, with a total of three doses.

For the MC38 colorectal tumor model, 5 × 10^5^ MC38 cells were injected subcutaneously into the right flank of mice in 100 μL PBS. Once the tumor volume reached approximately 200 mm^3^, the tumor-bearing mice were randomly assigned to treatment groups.

For IL-2 PEG conjugate treatment, 500 pmol of Cm-IL2 or Cm-proIL2 or PBS was injected intravenously at 2 day intervals, with a total of three doses.

For PD1-IL-2 bispecifics treatment, 250 or 500 pmol of PD1-Cm-IL2 or PD1-Cm-proIL2 or 500pmol of PD1-IL2m was injected intravenously at 2 day intervals, with a total of three doses.

Tumor size was monitored using the formula: length × (width)^2^/2. Mice were sacrificed when tumor volume exceeded 2000 mm^3^ or if body weight loss exceeded 20% during the study.

For PD1-Cm-IL2, another two doses of 500 pmol were injected intravenously at 2 day intervals a week after the initial treatment period.

For rechallenge studies, cured mice that remained tumor-free for approximately 45 days were reinoculated with 3 times the original dose of MC38 cells. As a comparison, 4 month-old naïve mice were injected with the same number of MC38 cells.

### Immune Subsets Analysis

MC38 tumor-bearing mice were euthanized 72 h after completing the three-dose treatment regimen. Tumors, dLNs, and spleens were collected for downstream analysis. Tumors from all groups were trimmed to a uniform size (∼150 mm^3^) and enzymatically dissociated in a digestion buffer containing collagenase IV (50 μg/mL, Sigma), DNase I (10 μg/mL, Sigma), collagenase I (0.3 mg/mL, Gibco), and 10 mM MgCl_2_ at 37 °C for 1 h. The resulting suspensions were passed through a 70 μm strainer, subjected to Percoll (Cytiva) density purification, and treated with ACK lysing buffer (Thermo Fisher Scientific) to remove residual red blood cells.

For spleens and dLNs, tissues were mechanically dissociated through 70 μm strainers; spleen samples additionally underwent ACK lysis.

Single-cell suspensions were stained with antibody panels and analyzed on an LSRFortessa flow cytometer (BD).

For tumor-specific and memory CD8^+^ T-cell profiling, the following antibodies were used: p15E tetramer-APC (1:200; MBL, TS-M507-2), anti-CD8-FITC (1:200; Invitrogen, MA516759), anti-CD45-BV650 (1:200; BioLegend, 109836), anti-CD3-PE/Cy7 (1:200; BioLegend, 100319), anti-CD44-PE (1:200; BioLegend, 103008), and anti-CD62L-APC/Cy7 (1:200; BioLegend, 104429). Cell viability was determined using Fixable Viability Dye eFluor 506 (Thermo Fisher).

Naive CD8^+^ T cells were gated as CD45^+^CD3^+^CD8^+^CD44^–^CD62L^+^, central memory cells as CD45^+^CD3^+^CD8^+^CD44^+^CD62L^+^, and effector memory cells as CD45^+^CD3^+^CD8^+^CD44^+^CD62L^–^.

For regulatory T-cell phenotyping, cells were fixed and permeabilized using the eBioscience Foxp3/Transcription Factor Staining Buffer Set (Invitrogen) according to the manufacturer’s instructions. The antibody panel included anti-CD45-FITC (1:400; BioLegend, 103107), anti-CD3-PE/Cy7 (1:200; BioLegend, 100320), anti-CD4-BV650 (1:200; Invitrogen, 64-0042-82), anti-CD8-APC (1:200; Invitrogen, 17-0083-81), anti-PD-1-APC/Cy7 (1:200; Invitrogen, 47-9985-82), anti-TIM-3-PE/Dazzle594 (1:200; BioLegend, 119748), anti-Foxp3-PE (1:200; Thermo Fisher, 12-5773-82), and anti-TCF-1-BV421 (1:100; Thermo Fisher, S33-966).

Viability was again assessed with Fixable Viability Dye eFluor 506.

### Single-Cell RNA Sequencing

The transcriptional profiles of tumor-infiltrating immune cells were analyzed in MC38 tumor-bearing mice treated with PBS, Cm-IL2, or Cm-proIL2. C57BL/6 mice were inoculated subcutaneously with MC38 colon adenocarcinoma cells and then randomized into three treatment groups for intravenous administration of the respective agents, as described in the in vivo efficacy study. Tumors were excised on day 2 post-treatment, enzymatically dissociated into single cells, and the live CD45^+^ immune cell fraction was subsequently isolated by flow-cytometry-based high-purity sorting (FACS). For single-cell RNA sequencing, barcoded libraries were prepared from the sorted cells. Briefly, this process involved droplet encapsulation with barcoded beads, emulsion breakage, bead collection, and reverse transcription. The synthesized cDNA was then amplified, purified, and used to generate indexed sequencing libraries following the manufacturer’s protocol. Library quality was evaluated by measuring concentration with the Qubit ssDNA Assay Kit (Thermo Fisher Scientific) and assessing size distribution on an Agilent Bioanalyzer 2100 system. The final libraries were subjected to paired-end sequencing on a DNBSEQ-T7 system (Annoroad Gene Technology Co., Ltd.). The sequencing output comprised a 30-base Read 1, which included a 10 bp cell barcode 1, a 10 bp cell barcode 2, and a 10 bp unique molecular identifier (UMI); a 100-base Read 2, corresponding to the gene sequence; and an additional 10-base read used for sample indexing.

### In Vivo Safety Evaluation

PD1-Cm-IL2 or PD1-Cm-proIL2 (500 pmol) or PBS was given intravenously into MC38-tumor-bearing C57BL/6J mice after tumor volumes reached approximately 200 mm^3^. Blood was collected 4 days after injection for complete blood count analysis and CK/LDH measurement. Complete blood counts were performed by using a Mindray BC-5000 Vet analyzer. Mice were euthanized on day 7 after treatment, and major organs were harvested for histological evaluation following H&E staining.

## Supplementary Material



## Data Availability

All data reported in this paper will be shared by the lead contact upon request. scRNA-seq data are deposited at GSA (https://ngdc.cncb.ac.cn/gsa/): CRA029360 and CRA029361. This study did not generate any unique codes. All other data can be made available from the authors upon reasonable request.

## Notes

Supplementary figures covering linker synthesis and protein conjugation, in vitro characterization, immunological analysis, and safety evaluations and supplementary data tables (PDF)
