# Supplementary material for: A Tumor-Microenvironment-Responsive Chemically Masked IL‑2 Prodrug Potentiates PD‑1 Checkpoint Blockade and Elicits Robust Antitumor Efficacy
Source: ACS Cent Sci. 2026 Aug 5;12(8):1223–36. doi: 10.1021/acscentsci.6c00344 (PMC13523670; doi:10.1021/acscentsci.6c00344)
Supplement: Supplementary file 1 [file oc6c00344_si_001.pdf]

Supplementary Information For

A Tumor Microenvironment Responsive Chemically Masked IL-2 Prodrug Potentiates PD-1 Checkpoint Blockade and Elicits Robust Antitumor Efficacy

Linzhi Tan[1,6], Yunwen Yang[2,6], Kerui Zhao[1], Jingjing Zhou[1], Xin Wang[1], Xingyu Zhao[2], Qiang Lu[1], Junyu Lin[2], Yijie Zhao[4], Yushun Tian[5], Yong Wang[1,*], Chao Zhong[2,*] & Tao Liu[1,3,*]

[1]State Key Laboratory of Natural and Biomimetic Drugs, Chemical Biology Center, Department of Molecular and Cellular Pharmacology, School of Pharmaceutical Sciences, Peking University Third Hospital, Institute of Advanced Clinical Medicine, Peking University, Beijing 100191, China

[2] Department of Immunology, School of Basic Medical Sciences, NHC Key Laboratory of Medical Immunology, Medicine Innovation Center for Fundamental Researches on Major Immunology-Related Diseases, Peking University, Beijing 100191, China.

[3]PKUHSC-Beijing Huida Cell Technology CO., Ltd Joint Laboratory for Codon Innovation, Beijing 100191, China

[4]Department of Laboratory Animal Science, Peking University Health Science Center, Beijing 100191, China

[5]Beijing Huida Cell Technology Co., Ltd., Beijing 100176, China

[6]These authors contributed equally: Linzhi Tan, Yunwen Yang.

[*]Correspondence to:

yong_wang@pku.edu.cn; zhongc@pku.edu.cn; taoliupku@pku.edu.cn

Contents

Supporting Figures (S1-S10) ……………………………….………….…………...S3

Supporting Tables (S1-S3)……………………………………………………………S15

References…………………………………………..……………………..S17

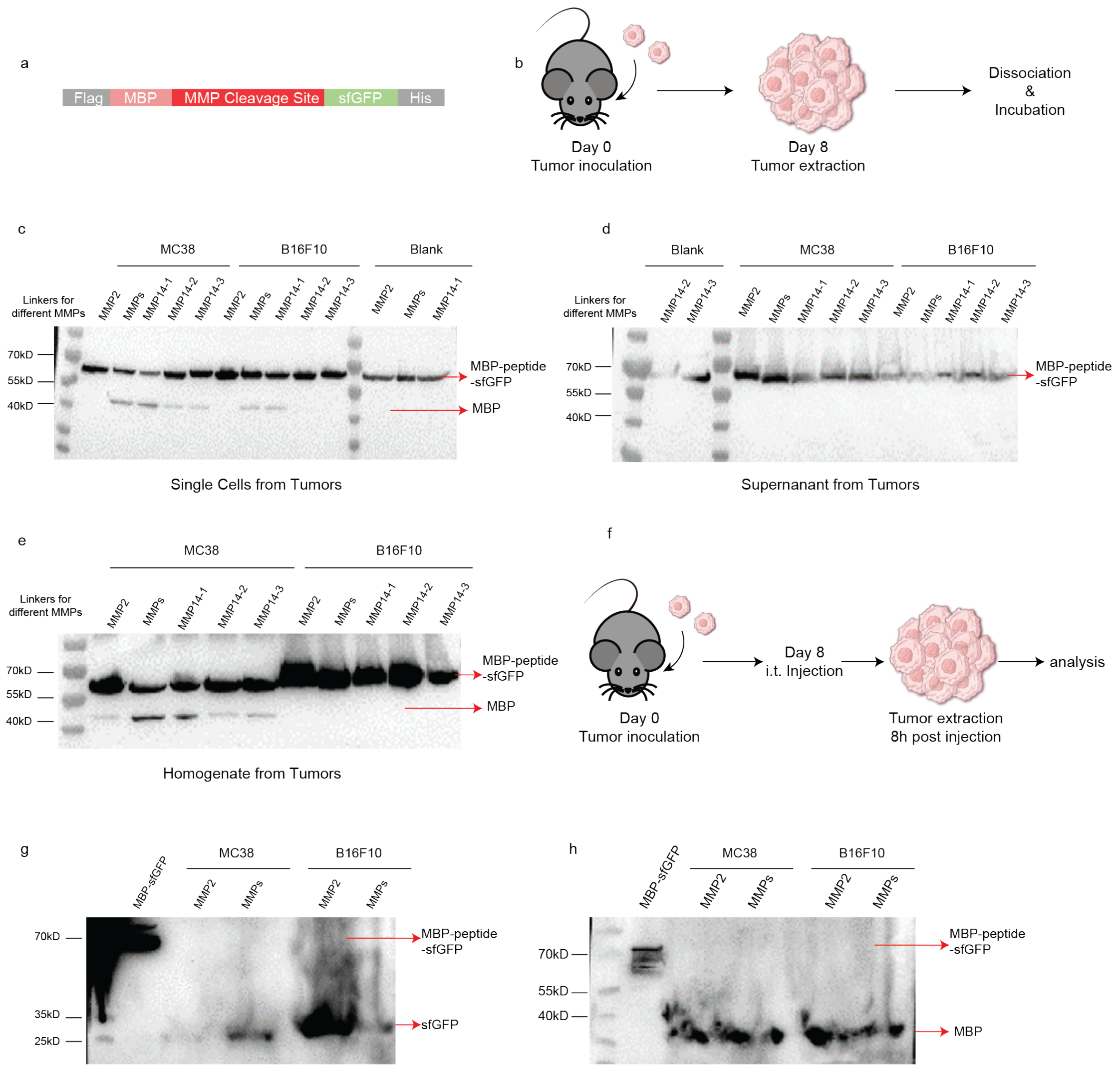

Figure S1. Screening of MMP-cleavable linkers for proIL-2 development. a, Model protein used for effective linker screening. **b**, Schematic of the in vitro screening workflow. Tumor cells were implanted subcutaneously, and tumors were harvested once established. **c-e,** In vitro cleavage was evaluated in c) single-cell suspensions, d) supernatants, and e) tumor homogenates. f, Schematic of the in vivo screening strategy. Each panel represents an MBP–peptide–sfGFP fusion protein containing a distinct MMP-cleavable linker. Linkers tested for MMP susceptibility included GSGPLGLAGGSG (MMP2) [1], GSGVPLSLYSGGSG (MMPs) [2], GSGSGARYRWLTAGSG (MMP14-1), GSGSGRSYAILTAGSG (MMP14-2), and GSGSGFIANPVTAGSG (MMP14-3) [3], with the underlined residues indicating the MMP recognition sites. MBP-peptide-sfGFP fusion proteins were intratumorally injected into established tumors. Tumors were harvested 8 h post injection, homogenized, and subjected to western blot analysis. Two candidate linkers, MMP2 and MMPs, were selected for in vivo validation. **g-h**, In vivo cleavage was confirmed by immunoblotting with **g)** anti-His and **h)** anti-Flag antibodies.

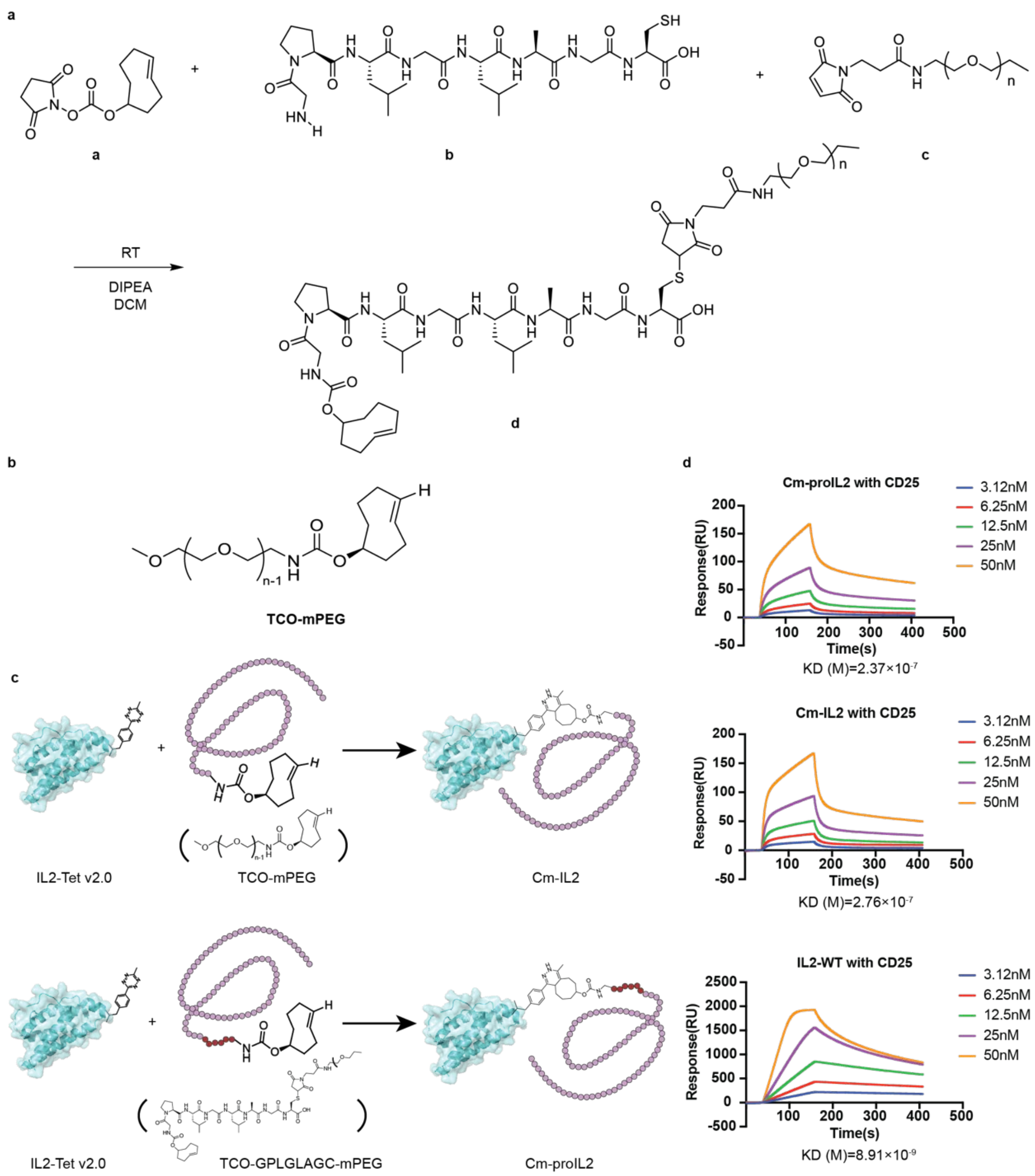

Figure S2. a, Synthesis of TCO-PLGLAG-PEG20K. A total of 32.15 g of methyl-PEG20000-maleimide (**a**) (1.61 mmol; Confluore Life Science Accelerator) was weighed, dissolved in approximately 2 mL dichloromethane, and transferred to a reaction flask for subsequent use. Then, 2.2 mg of a peptide (**b**) synthesized by GenScript (sequence: GPLGLAGC, in which the terminal Gly and Cys residues were used for conjugation to TCO and PEG, respectively) was dissolved in 44 μL dimethyl sulfoxide and immediately added to the reaction mixture. Subsequently, 0.537 mg of (4E)-TCO-NHS ester (**c**) (0.00201 mmol; Confluore Life Science Accelerator) was introduced. The mixture was stirred at room temperature for 8 h. Afterward, 0.39 g of N,N-diisopropylethylamine (0.003015 mmol) was added, and the reaction was continued for an additional 2 h at room temperature. Upon completion, the reaction mixture was concentrated and dried, yielding the white solid product TCO-PLGLAG-

PEG20K (**d**). **b,** Chemical structure of an uncleavable TCO-mPEG linker used for the construction of Cm-IL2. **c,** Schematic of Cm-IL2 and Cm-proIL2 constructs. **d,** SPR analysis of binding between IL-2 constructs and human IL-2Rα.

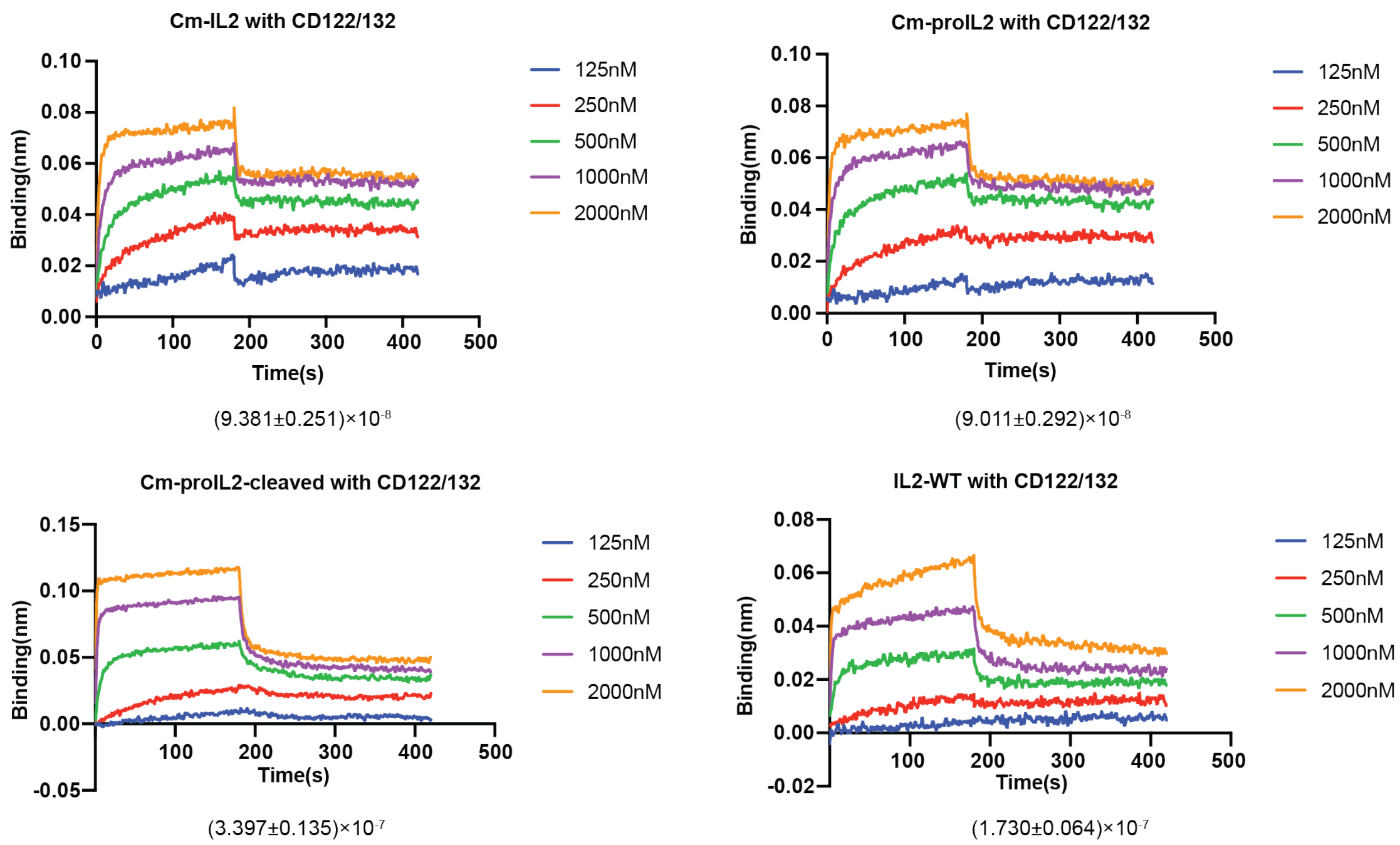

Figure S3. Characterization of IL-2–PEG constructs. Binding kinetics of Cm-IL2, Cm-proIL2, MMP2-cleaved Cm-proIL2, and IL-2-WT to IL-2Rβγ complexes measured by BLI using AR2G sensors.

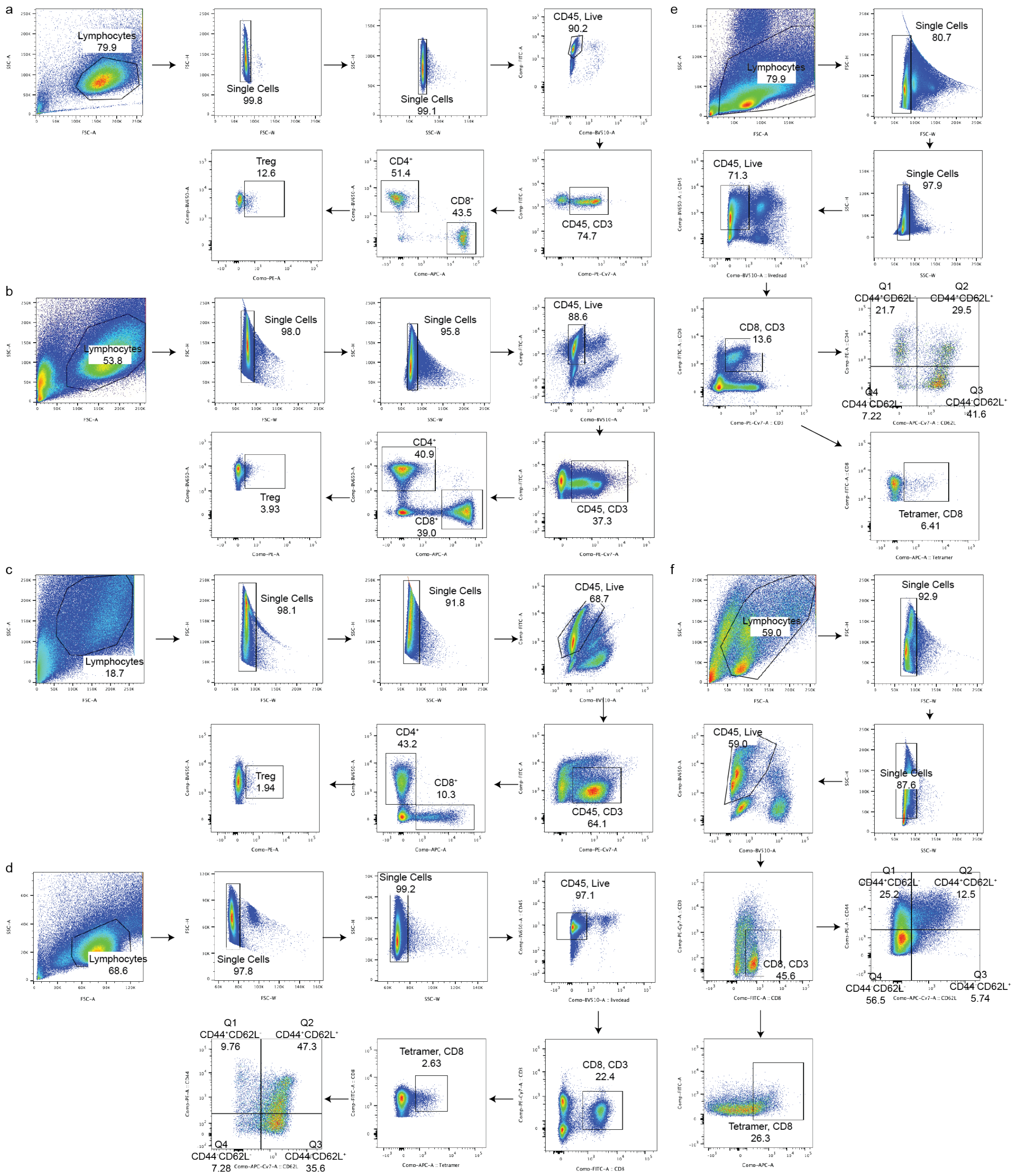

Figure S4. Flow cytometric characterization of immune cell subsets following treatment with IL-2–based constructs. a-c, Representative gating strategies for identification of CD3⁺, CD4⁺, and CD8⁺ T cells, as well as Tregs, in **a)** dLNs, **b)** spleens and **c)** tumors. Related to **Fig. 3d-e** and **Fig. 6b. d-f,** Representative gating strategies for identification of CD8⁺ antigen-specific T cells and memory T cells, in **d)** dLNs, **e)** spleens and **f)** tumors. Naïve T cells were defined as CD62⁺CD44⁻ and central memory T cells (Tcm) as CD62L⁺CD44⁺. Related to **Fig. 3f-j**, **Fig. 6c** and **Fig S5**.

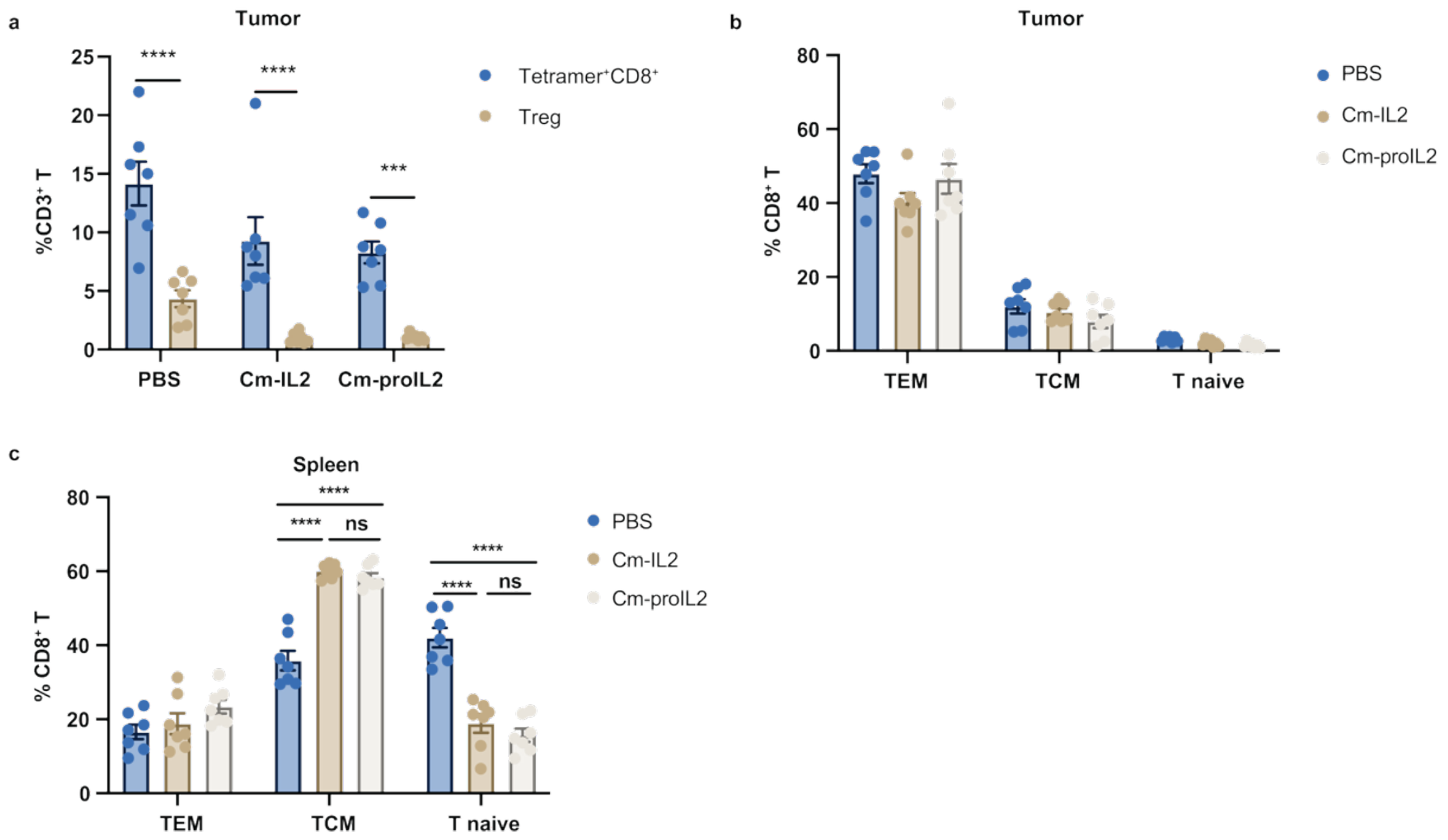

Figure S5. Characterization of T-cell subsets in tumors and spleens. a, Bar graphs showing the frequencies of CD8+Tetramer+ T cells and Tregs within CD3+ T cells of PBS, Cm-IL2 and Cm-proIL2 treated mice in tumor microenvironment. **b-c,** Bar graphs showing the frequencies of effector memory T cells (TEM), central memory T cells (TCM), and naïve T cells (T naïve) within the $CD8^+$ T-cell compartment from mice treated with PBS, Cm-IL2, or Cm-proIL2 in (b) tumors and (c) spleens. Statistical significance was determined using unpaired two-tailed t tests. ****P < 0.0001; ns, not significant.

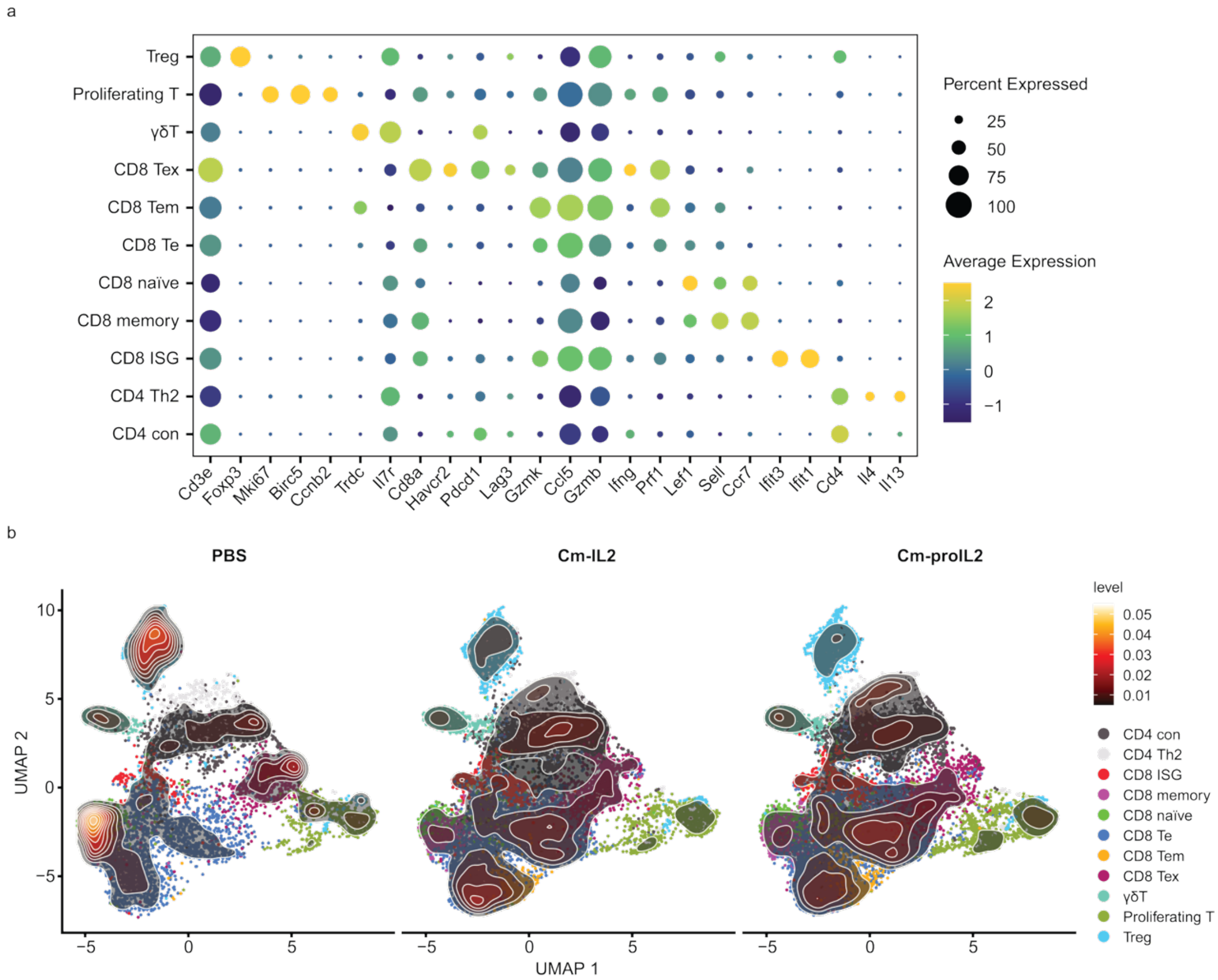

Figure S6. scRNA-seq analysis of Cm-IL2 and Cm-proIL2 treatment. a, Dot plot showing marker gene expression for each UMAP cluster identified in **Fig.4a**. **b,** UMAP contour maps overlaid by treatment group.

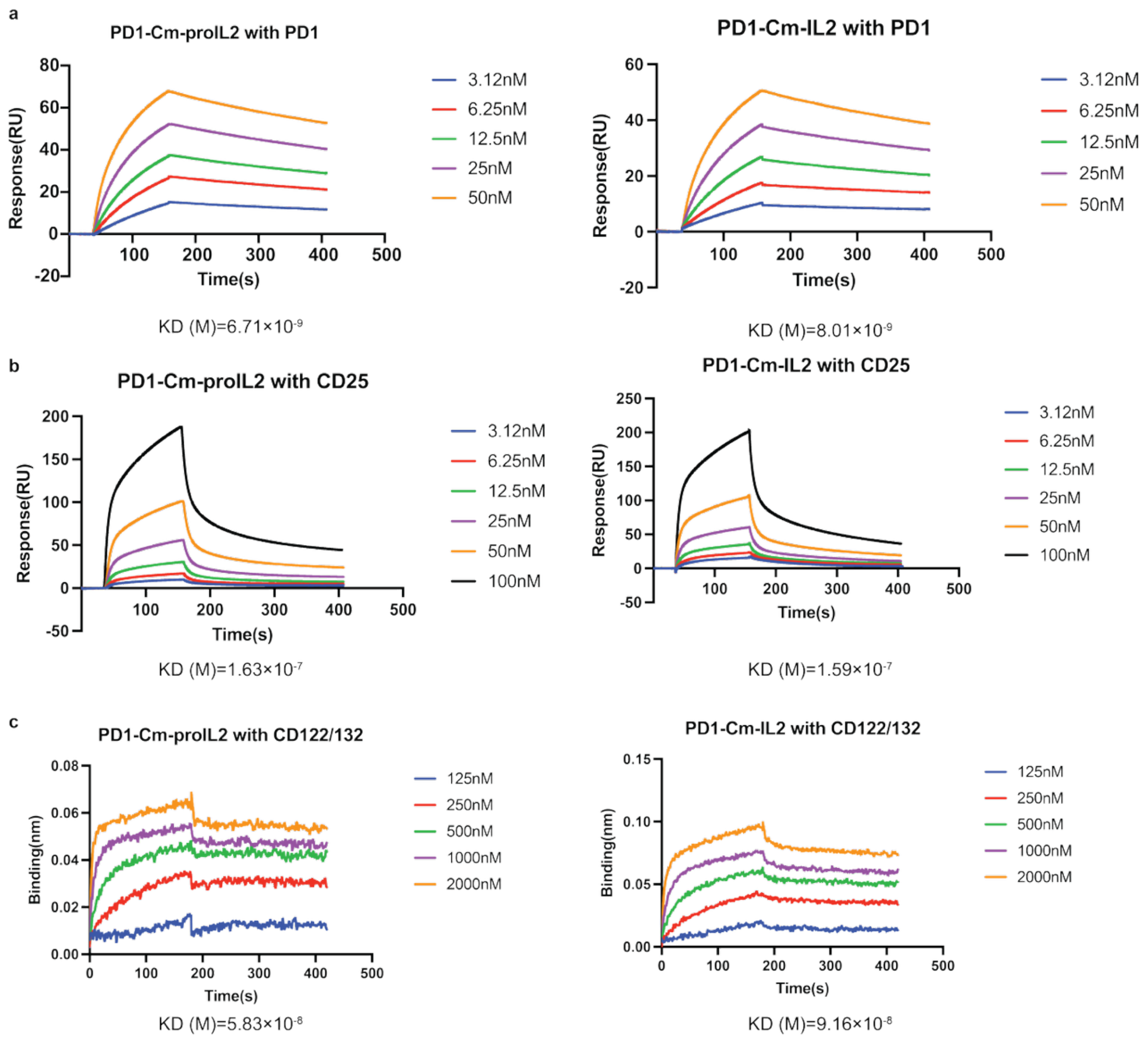

Figure S7. **Binding characterization of PD1-Cm-proIL2 and PD1-Cm-IL2. a**, Binding of PD1-Cm-proIL2 and PD1-Cm-IL2 to human PD-1 (*hu*PD-1), determined by surface plasmon resonance (SPR). **b**, Binding of PD1-Cm-proIL2 and PD1-Cm-IL2 to human CD25 (*hu*IL-2Rα), determined by SPR. **c**, Binding of PD1-Cm-proIL2 and PD1-Cm-IL2 to human CD122/CD132 (*hu*IL-2Rβγ), determined by BLI.

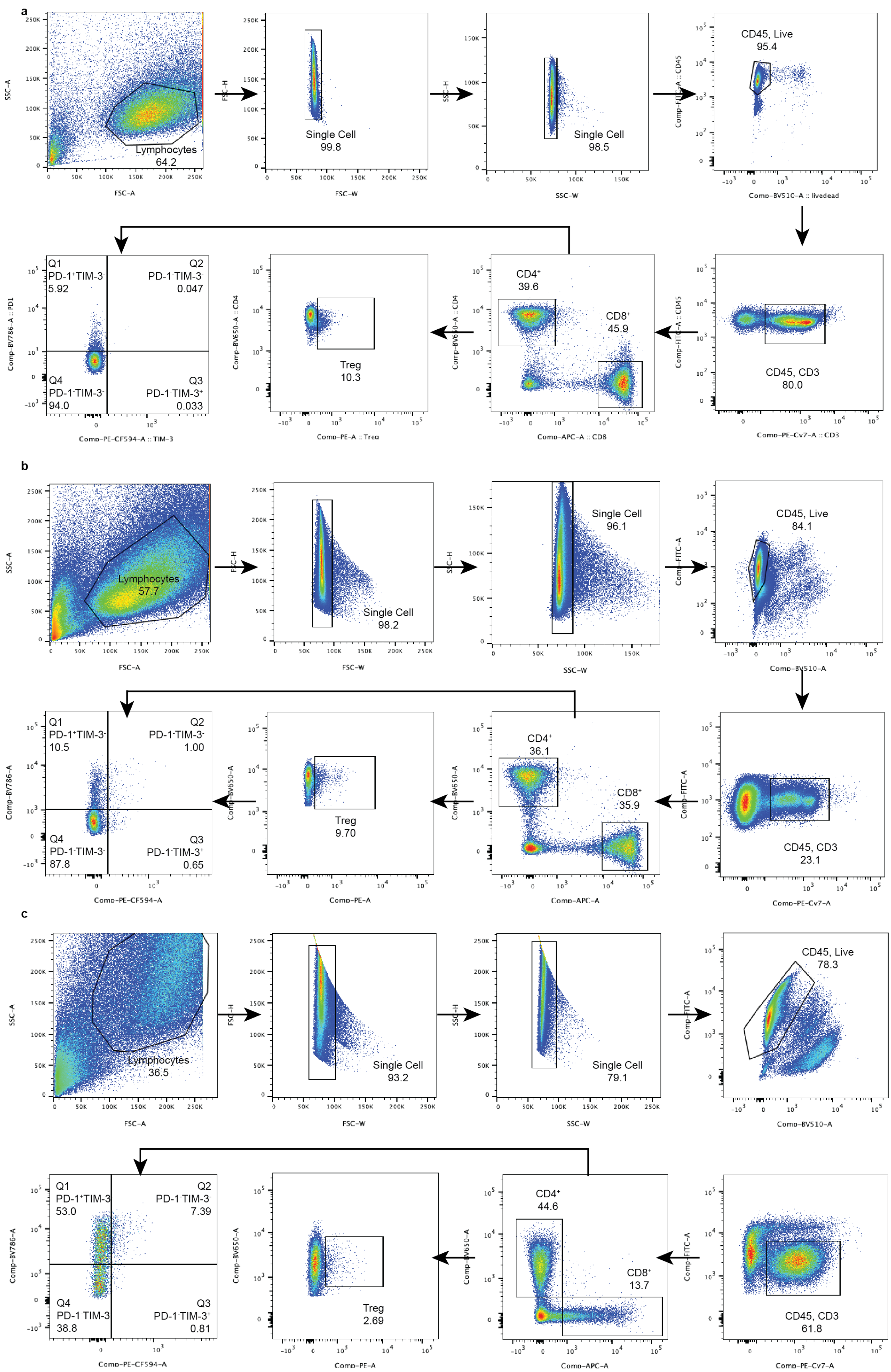

Figure S8. Flow cytometric characterization of immune subsets following PD1-Cm-IL2 and PD1-Cm-proIL2 treatment. Gating strategy for T-cell subset analysis

in a) dLNs, b) spleens and c) tumors, including $CD3^+$, $CD4^+$, and $CD8^+$ T cells, Tregs, and exhaustion marker profiling. Related to **6d-e.**

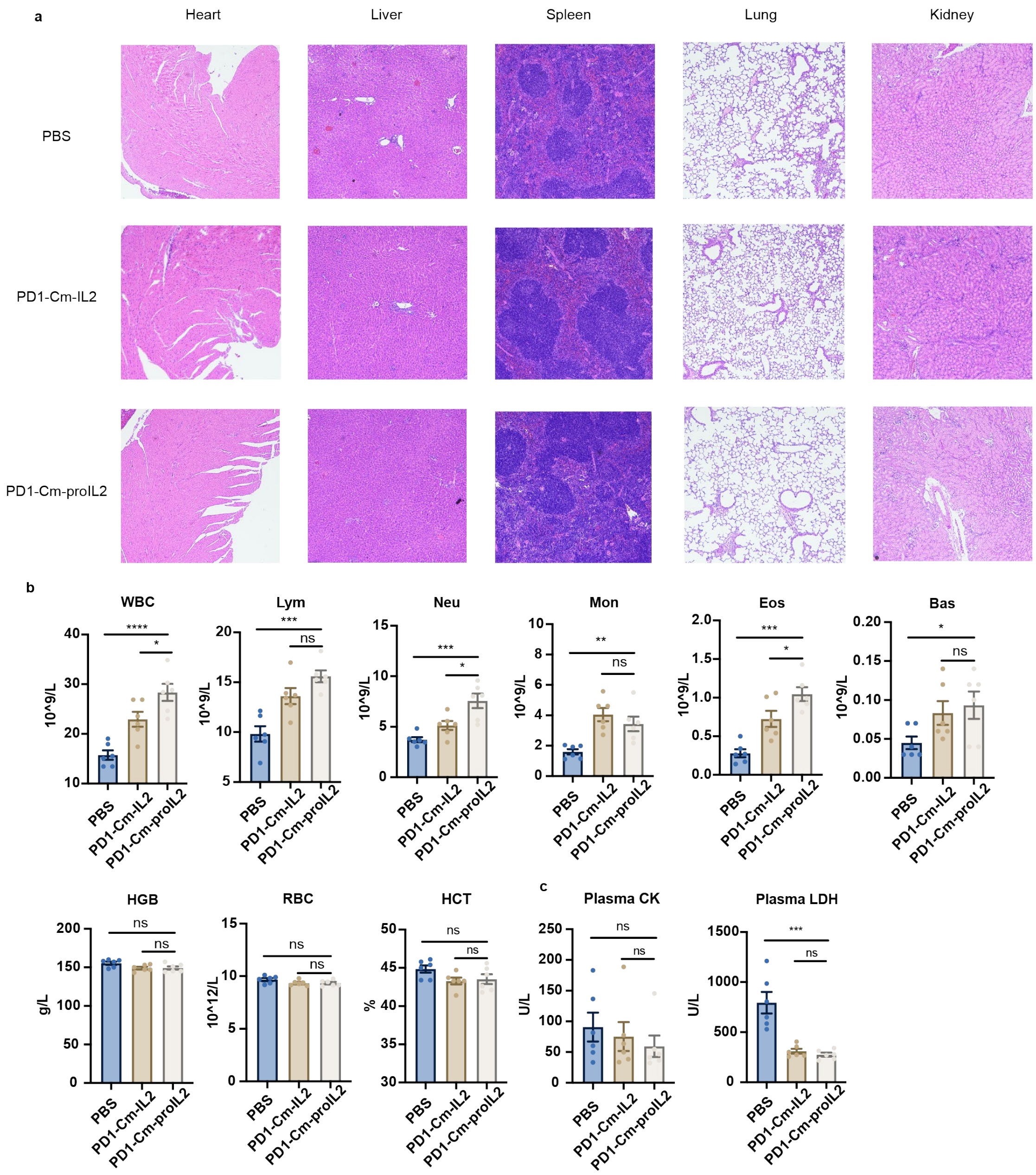

Figure S9. Safety evaluation of single-dose treatment of PD1-Cm-IL2 and PD1-Cm-proIL2. a, Representative hematoxylin and eosin (H&E)–stained sections of major organs. Representative H&E staining of heart, liver, spleen, lung, and kidney tissues from mice treated with PBS, PD1-Cm-IL2, or PD1-Cm-proIL2. **b-c**, **b)** Complete blood count (CBC) and **c)** plasma creatine kinase (CK) and lactate dehydrogenase (LDH) analyses of mice treated with PBS, PD1-Cm-IL2 or PD1-Cm-proIL2. The data are presented as the MEAN ± SEM. *P<0.05, **P<0.01, ***P<0.001, ****P<0.0001.

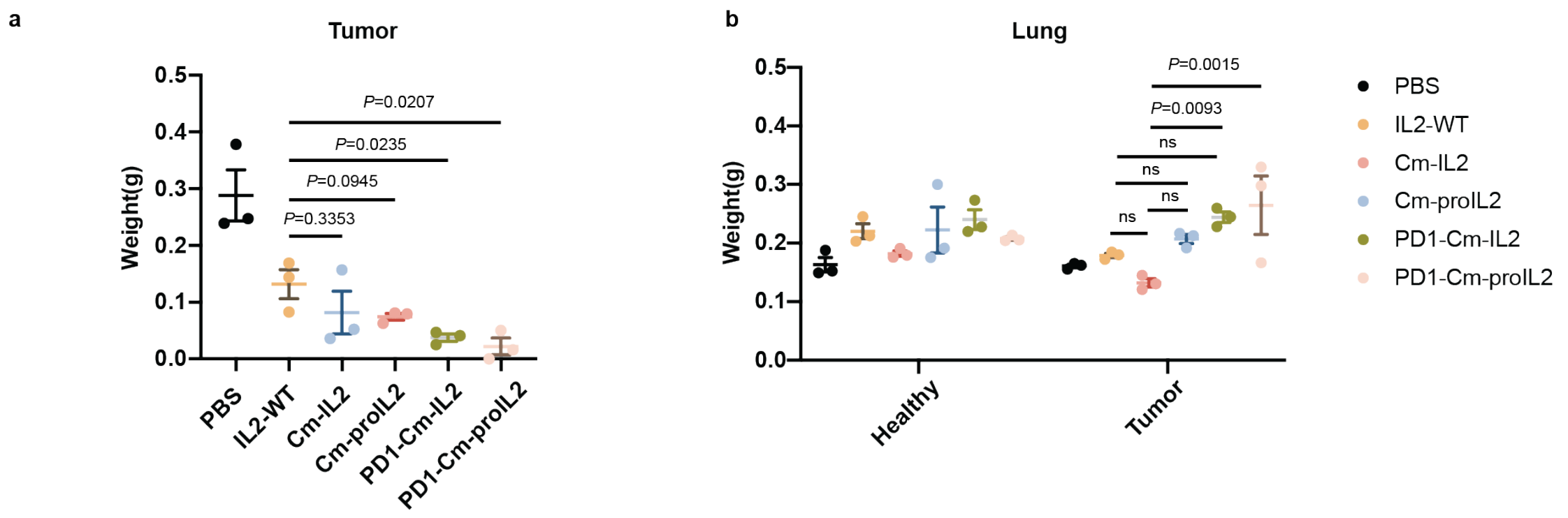

Fig S10. Systematic safety evaluation of IL-2-based constructs. a, Tumor weights at the end of the dosing period. **b**, Lung weights measured at the end of the dosing period.

Table S1. Proteins and related sequence used in this study

Name (All in pET22B (+) backbone)	Sequence
T7 promoter	TAATACGACTCACTATAGG
IL-2 (human)	APTSSSTKKTQLQLEHLLLDLQMILN<br>GINNYKNPKLTRMLTFKFYMPKKATE<br>LKHLQCLEEELKPLEEVLNLAQSKNF<br>HLRPRDLISNINVIVLELKGSETTFMC<br>EYADETATIVEFLNRWITFCQSIISTLTL<br>EHHHHHH
Nb11 (anti-PD1 nanobody)	From WO2024083988A1
MBP	GDYKDDDDKGKIEEGKLVIWINGDK<br>GYNGLAEVGKKFEKDTGIKVTVEHP<br>DKLEEKFPQVAATGDGPDIIFWAHDR<br>FGGYAQSGLLAEITPDKAFQDKLYPF<br>TWDAVRYNGKLIAYPIAVEALSLIYN<br>KDLLPNPPKTWEEIPALDKELKAKGK<br>SALMFNLQEPYFTWPLIAADGGYAFK<br>YENGKYDIKDVGVDNAGAKAGLTFL<br>VDLIKNKHMNADTDYSIAEAAFNKG<br>ETAMTINGPWAWSNIDTSKVNYGVT<br>VLPTFKGQPSKPFVGVLSAGINAASP<br>NKELAKEFLENYLLTDEGLEAVNKDK<br>PLGAVALKSYEEELVKDPRIAATMEN<br>AQKGEIMPNIPQMSAFWYAVRTAVIN<br>AASGRQTVDEALKDAQT
sfGFP	SKGEELFTGVVPILVELDGDVNGHKF<br>SVRGEGEGDATNGKLTLKFICTTGKL<br>PVPWPTLVTTLTYGVQCFSRYPDHMK<br>RHDFFKSAMPEGYVQERTISFKDDGT<br>YKTRAEVKFEGDTLVNRIELKGIDFK<br>EDGNILGHKLEYNFNSHNVYITADKQ<br>KNGIKANFKIRHNVEDGSVQLADHY<br>QQNTPIGDGPVLLPDNHYLSTQSVLS<br>KDPNEKRDHMVLLEFVTAAGITHGM<br>DELYK
MMP2[1]	PLGLAG
MMPs[2]	VPLSLYSG
MMP14-1[3]	SGARYRWLTA
MMP14-2[3]	SGRSYAILTA
MMP14-3[3]	SGFIANPVTA

Table S2. Binding affinities of IL-2–PEG conjugates to murine IL-2Rα

	Cage/KD(M)	Decage/KD(M)	Fold Change
P34	$(1.04\pm0.41)\times10^{-6}$	$(2.92\pm0.23)\times10^{-7}$	3.56
K35	$(4.05\pm0.029)\times10^{-6}$	$(4.23\pm0.76)\times10^{-7}$	9.57
F42	NA	$(1.04\pm0.057)\times10^{-6}$	-
Y45	$(2.58\pm0.161)\times10^{-6}$	$(1.09\pm0.76)\times10^{-6}$	2.37
E61	$(8.04\pm0.68)\times10^{-6}$	$(4.91\pm0.029)\times10^{-6}$	1.64
E62	NA	$(1.87\pm0.056)\times10^{-6}$	-
K64	$(3.77\pm0.77)\times10^{-6}$	$(2.41\pm0.058)\times10^{-7}$	15.6
E67	$(9.79\pm0.35)\times10^{-7}$	$(1.12\pm0.061)\times10^{-6}$	1.14
Q74	$(3.51\pm0.36)\times10^{-6}$	$(2.626\pm0.55)\times10^{-7}$	13.4

***NA, not available**

Table S3. Antibodies used in this study

Antibodies (Clone)	Source	Catalog
Flow cytometry		
APC anti-human pSTAT5 (A17016B.Rec)	Biolegend	936906
T-Select H-2K^b MuLV p15E Tetramer-KSPWFTTL-APC	MBL	TS-M507-2
FITC anti-mouse CD8 (KT15)	Invitrogen	MA-516759
BV650 anti-mouse CD45 (104)	Biolegend	109836
PE/CY7 anti-mouse CD3 (145-2C11)	Biolegend	100319
PE anti-mouse CD44 (1M7)	Biolegend	103008
APC/CY7 anti-mouse CD62L (MEL-14)	Biolegend	104429
FITC anti-mouse CD45 (30-F11)	Biolegend	103107
BV650 anti-mouse CD4 (RM4-5)	Invitrogen	64-0042-82
APC anti-mouse CD8 (H35-17.2)	Invitrogen	17-0083-81
APC/CY7 anti-mouse PD-1 (J43)	Invitrogen	47-9985-82
BV785 anti-mouse PD-1 (29F.1A12)	Biolegend	135225
PE/Dazzle594 anti-mouse TIM-3 (RMT3-23)	Biolegend	119748
PE anti-mouse Foxp3 (FJK-16s)	Thermo Fisher	12-5773-82
BV421 anti-mouse TCF-1 (S33-966)	BD Biosciences	566692
ELISA		
Anti-IL-2 Antibody (HRP), Rabbit Polyclonal	Sinobiological	11848-RP01-H

References

1. Trang, V. H. *et al.* A coiled-coil masking domain for selective activation of therapeutic antibodies. *Nature Biotechnology* **37**, 761–765 (2019).
2. Mansurov, A. *et al.* Masking the immunotoxicity of interleukin-12 by fusing it with a domain of its receptor via a tumour-protease-cleavable linker. *Nature Biomedical Engineering* **6**, 819–829 (2022).
3. Hsu, E. J. *et al.* A cytokine receptor-masked IL2 prodrug selectively activates tumor-infiltrating lymphocytes for potent antitumor therapy. *Nature Communications* **12**, 2768 (2021).
